# Mining Translation Inhibitors by a Unique Peptidyl-Aminonucleoside Synthetase Reveals Cystocin Biosynthesis and Self-Resistance

**DOI:** 10.3390/ijms252312901

**Published:** 2024-11-30

**Authors:** Vera A. Alferova, Polina A. Zotova, Anna A. Baranova, Elena B. Guglya, Olga A. Belozerova, Sofiya O. Pipiya, Arsen M. Kudzhaev, Stepan E. Logunov, Yuri A. Prokopenko, Elisaveta A. Marenkova, Valeriya I. Marina, Evgenia A. Novikova, Ekaterina S. Komarova, Irina P. Starodumova, Olga V. Bueva, Lyudmila I. Evtushenko, Elena V. Ariskina, Sergey I. Kovalchuk, Konstantin S. Mineev, Vladislav V. Babenko, Petr V. Sergiev, Dmitrii A. Lukianov, Stanislav S. Terekhov

**Affiliations:** 1 Shemyakin-Ovchinnikov Institute of Bioorganic Chemistry, Miklukho-Maklaya 16/10, 117997 Moscow, Russia; alferovava@gmail.com (V.A.A.); anjabaranowa@list.ru (A.A.B.); eguglya@gmail.com (E.B.G.); o.belozyorova@gmail.com (O.A.B.); pipiyasofiya@ibch.ru (S.O.P.); kudzhaev_arsen@mail.ru (A.M.K.); stepa.2003@bk.ru (S.E.L.); tetrahydrofuran@mail.ru (Y.A.P.); marenkova.lizzz@gmail.com (E.A.M.); iri-starodumova@yandex.ru (I.P.S.); xerx222@gmail.com (S.I.K.); mineev@nmr.ru (K.S.M.); 2 Department of Chemistry, Lomonosov Moscow State University, 119992 Moscow, Russia; zotova-polina98@mail.ru (P.A.Z.); ymmo@mail.ru (V.I.M.); zhizhenzha@gmail.com (E.A.N.); petya@genebee.msu.ru (P.V.S.); dmitrii.a.lukianov@gmail.com (D.A.L.); 3 A.N. Belozersky Institute of Physico-Chemical Biology, Lomonosov Moscow State University, 119991 Moscow, Russia; ekaandreyanova@yandex.ru; 4 All-Russian Collection of Microorganisms (VKM), Pushchino Scientific Center for Biological Research, Russian Academy of Sciences, 142290 Pushchino, Russia; oboueva@mail.ru (O.V.B.); lie99@mail.ru (L.I.E.); ariskina@pbcras.ru (E.V.A.); 5 Lopukhin Federal Research and Clinical Center of Physical-Chemical Medicine, Malaya Pirogovskaya Str. 1a, 119435 Moscow, Russia; daniorerio34@gmail.com; 6 Center for Molecular and Cellular Biology, 121205 Moscow, Russia

**Keywords:** puromycin, cystocin, aminonucleoside antibiotics, resistance, genome mining, antimicrobial activity, cytotoxicity

## Abstract

Puromycin (Puro) is a natural aminonucleoside antibiotic that inhibits protein synthesis by its incorporation into elongating peptide chains. The unique mechanism of Puro finds diverse applications in molecular biology, including the selection of genetically engineered cell lines, in situ protein synthesis monitoring, and studying ribosome functions. However, the key step of Puro biosynthesis remains enigmatic. In this work, pur6-guided genome mining is carried out to explore the natural diversity of Puro-like antibiotics. The diversity of biosynthetic gene cluster (BGC) architectures suggests the existence of distinct structural analogs of puromycin encoded by pur-like clusters. Moreover, the presence of tRNA^Cys^ in some BGCs, i.e., *cst*-like clusters, leads us to the hypothesis that Pur6 utilizes aminoacylated tRNA as an activated peptidyl precursor, resulting in cysteine-based analogs. Detailed metabolomic analysis of *Streptomyces* sp. VKM Ac-502 containing *cst*-like BGC revealed the production of a cysteinyl-based analog of Puro—cystocin (Cst). Similar to puromycin, cystocin inhibits both prokaryotic and eukaryotic translation by the same mechanism. Aminonucleoside N-acetyltransferase CstC inactivated Cst, mediating antibiotic resistance in genetically modified bacteria and human cells. The substrate specificity of CstC originated from the steric hindrance of its active site. We believe that novel aminonucleosides and their inactivating enzymes can be developed through the directed evolution of the discovered biosynthetic machinery.

## 1. Introduction

Puromycin (Puro) is a well-known aminonucleoside antibiotic that blocks protein synthesis [1]. Puro mimics the aminoacyl end of tRNA entering the ribosome A site. Puro is transferred to the growing peptide chain, causing the formation of a puromycylated nascent chain and premature chain release [2]. This mode of action makes Puro a valuable tool for various applications in molecular biology research. For example, puromycylation, typically detected by anti-puromycin antibodies, is used for monitoring protein synthesis in living cells [3,4] and to study the subcellular localization of translating ribosomes [5]. Puro-induced release of ribosome-bound nascent chains is employed in various studies of ribosome functions, including ribosome profiling [5,6] and in situ translation [7,8]. Additionally, puromycin coupling is a crucial step for mRNA display technology [9].

Puro was isolated from *Streptomyces alboniger*, and its biosynthesis pathway attracted significant attention [10,11,12,13,14,15,16,17,18,19,20,21,22]. After initial identification of the *pur* biosynthetic gene cluster (BGC) [11], individual enzymes were studied in detail, including NAD-dependent ATP dehydrogenase Pur10 [10], tyrosinyl-aminonucleoside synthetase Pur6 [12], monophosphatase Pur3 [21], O-methyltransferase [17], and resistance-mediating enzymes Pur8 [13], PAC [14,22], Pur7 [20], and N-acetylpuromycin N-acetylhydrolase [15]. Moreover, the regulation mechanism of Puro production was investigated [16,18,19]. The self-resistance mechanism involving the acetylation of the antibiotic into inactive form by the puromycin N-acetyltransferase (PAC) [22] found practical application. This mechanism underlies a widely used cell line selection system [5,23]. Despite its widespread use in biotechnology, the key step in puromycin biosynthesis remains elusive [11]. In this work, we investigate the diversity of Puro-like BGCs to reveal the chemical space of Puro analogs and biosynthetic mechanisms behind aminonucleoside assembly.

## 2. Results

### 2.1. Genome Mining for Puromycin-like Antibacterials

**Figure 1:**
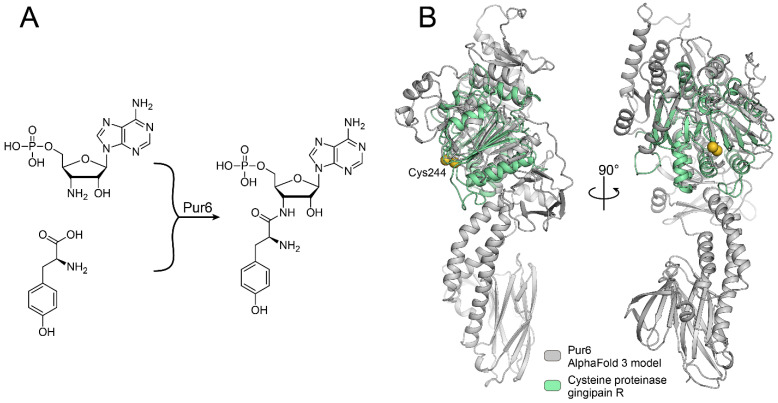
The key step of puromycin biosynthesis is mediated by the unique enzyme Pur6. (**A**) A schematic representation of the reaction catalyzed by Pur6 that was proposed in [11]. (**B**) Pur6 structure model generated by AlfaFold3 (grey) aligned with cysteine proteinase (green) gingipain R (PDB ID: 1CVR). Essential catalytic Cys244 residue of gingipain R and Cys326 residue of Pur6 are indicated as yellow spheres.

The BGC of puromycin provides clues for puromycin biosynthesis. However, we are still far from a detailed mechanism of it. The most uncommon step in puromycin biosynthesis is the coupling of amino acid tyrosine and nucleotide moieties (Figure 1A). According to puromycin BGC, this step must be mediated by a unique enzyme, Pur6 [11]. Pur6 is particularly interesting since this 84 kDa protein has no known conserved domains that could be identified by its sequence homology. Moreover, its AlfaFold 3 structure model has only weak and partial structural homology with cysteine proteinase gingipain R, which is the most proximate structural homolog known yet (Figure 1B).

RMSD between the 139–404 fragment of the Pur6 model and the 69–349 fragment of gingipain R is 2.14 Å. The Cys326 residue of Pur6 is located close to the active Cys244 residue that mediates the proteolytic activity of gingipain R, which indicates its potential involvement in the enzymatic reaction. However, the majority of Pur6 structures could not be assessed even by structural homology, which is outstanding for such a large enzyme.

**Figure 2:**
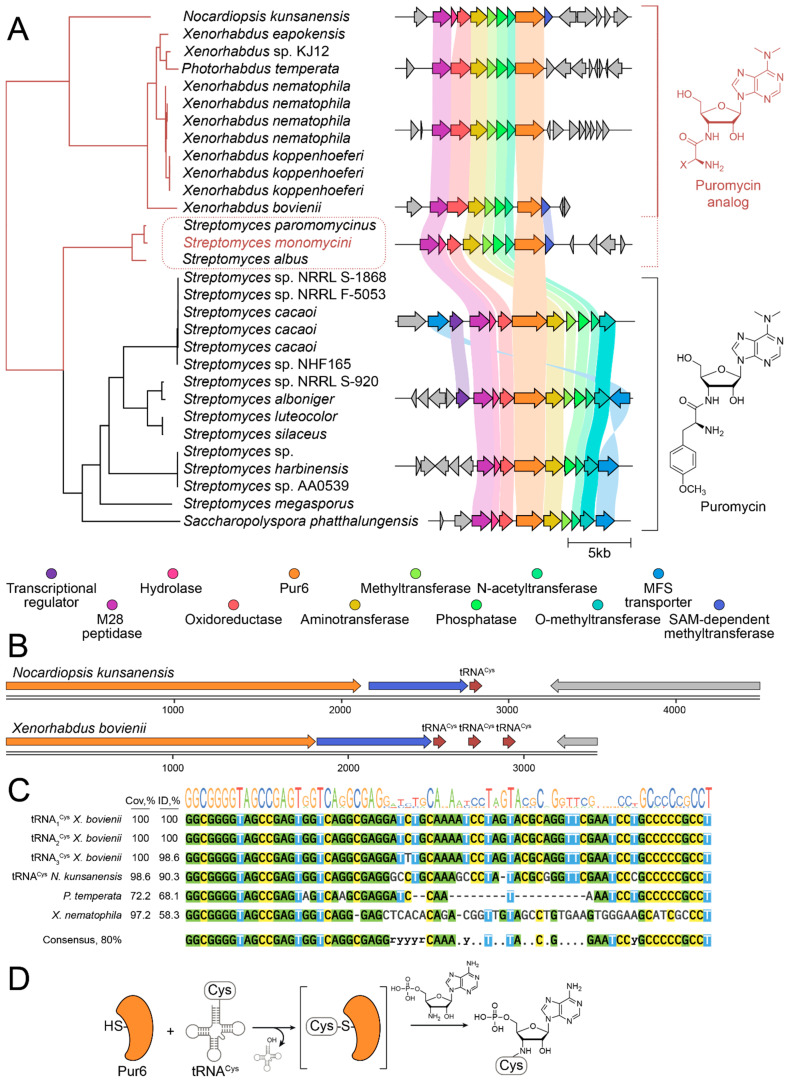
Pur6-guided genome mining revealed the biodiversity of puromycin analogs in *Streptomyces* and *Xenorhabdus*. (**A**) Pur6 and its homologs are strongly associated with BGC of puromycin-like secondary metabolites. The phylogenetic tree indicates the distance between Pur6 homologs in various bacteria. The corresponding genomic context and puromycin BGCs are colored with grey and rainbow, respectively. Putative BGCs associated with puromycin biosynthesis and biosynthesis of puromycin-like analogs are united by brackets and colored with black and red, respectively. Putative new analogs of puromycin in streptomycetes are highlighted with a red dotted line. (**B**) BGCs of puromycin-like secondary metabolites in *Nocardiopsis kunsanensis* and *Xenorhabdus bovienii* have cysteine tRNA. (**C**) Sequences of tRNA^Cys^ and similar RNA sequences encoded in the genomic context of BGCs of puromycin-like secondary metabolites. (**D**) Putative reaction scheme illustrating a potential working principle of Pur6-like enzymes.

To understand the diversity and potential catalytic mechanism of *pur6*-like enzymes, we performed genome mining of sequences similar to *pur6* (Figure 2). Pur6-like enzymes were strongly associated with puromycin BGCs in various *Streptomyces* species, including *S. alboniger*, *S. cacaoi*, *S. luteocolor*, *S. silaceus*, *S. harbinensis*, *S. megasporus*, and also in *Saccharopolyspora phatthalungensis*. While transcription regulation and transport mechanisms were often different, the core BGC architecture was generally the same (Figure 2A). Instead, an alternative BGC architecture and methyltransferase were detected for *S. monomycini*, *S. paromomycinus*, and *S. albus*. Moreover, this architecture was detected in *Nocardiopsis kunsanensis*, a member of a different actinomycete order, *Propionibacteriales*, and even in the gammaproteobacterium *Xenorhabdus bovienii*, which belongs to the phylogenetic branch very distant from actinomycetes. This may indicate both an alternative biosynthetic product and potential horizontal transfer between these species.

Incorporation of an amino acid residue in the biosynthetic product is common in the biosynthesis of secondary metabolites, especially those encoded by nonribosomal peptide synthetase (NRPS) BGCs. However, classical amino acid activation mechanisms such as standalone adenylation domains or ATP-binding domains were not detected in BGCs of puromycin-like secondary metabolites. Therefore, no catalytic activity responsible for the activation of the amino acid precursor was detected in both Pur6 and puromycin-like BGCs. In this regard, a particularly important question about the mechanism of the amino acid-nucleotide coupling reaction arises. A detailed analysis of the BGC genomic context revealed cysteine tRNA encoded at the end of puromycin-like BGCs in *N. kunsanensis* and *X. bovienii* (Figure 2B). Similar structures were detected at the end of puromycin-like BGCs in *Photorhabdus temperate* and other *Xenorhabdus*, including *Xenorhabdus nematophila* (Figure 2C). Hence, trying to find an activated amino acid precursor, we hypothesize that aminoacyl-tRNA may serve as an amino acid donor in the coupling reaction catalyzed by Pur6-like enzymes (Figure 2D). Taking into account structural homology with cysteine proteinases, we suggest that Pur6 uses aminoacyl-tRNA as a substrate, and Cys326 is involved in the active intermediate formation that simulates the common mechanism of acyl activation by the acyl carrier protein (ACP) domain in NRPSs and PKSs. If this hypothesis is correct, the biosynthetic product of puromycin-like BGCs in *N. kunsanensis*, *X. bovienii*, and *S. monomycini* should contain cysteinyl residue.

### 2.2. Cystocin Is a Biosynthetic Product of Puromycin-like BGC

**Figure 3:**
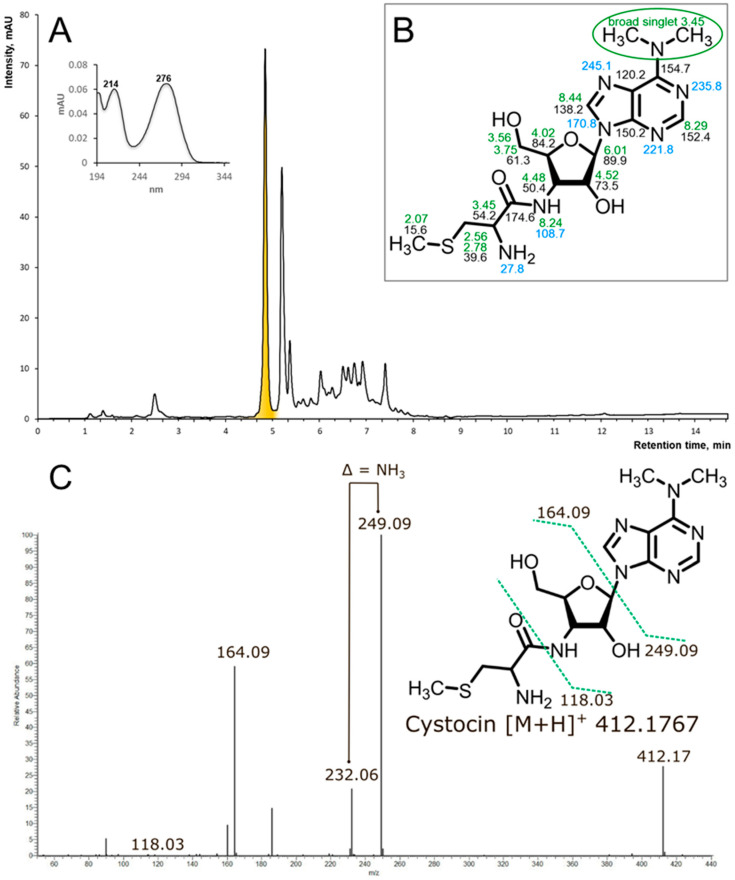
(**A**) rp-HPLC of the active fraction (Agilent HC-C18(2) 4.6 × 150 mm, 5 μm, eluent: solvent A—10 mM NH_4_OAc, pH 5, solvent B—MeCN, gradient elution from 15 to 90 solvent B for 10 min, flow rate 1.5 mL/min, UV 275 nm, sample volume 2 uL, active component is highlighted with yellow. (**B**) The structure of Cst with the indication of NMR chemical shifts in DMSO-d6 at 35 °C. Green numbers stand for the ^1^H chemical shifts, black—for the ^13^C, and blue—for ^15^N. Chemical shifts are provided in ppm, measured with respect to trimethylsylan as an external reference standard. ^13^C and ^15^N shifts were referenced indirectly, using the gyromagnetic ratios as implemented in Topspin software (Bruker Biospin Gmbh, Germany). (**C**) The positive mode HCD mass spectra of Cst and proposed fragmentation of the parent ion of the compound at *m*/*z* 412.17.

To investigate the exact biosynthetic product of puromycin-like BGCs in the aforementioned streptomycetes, we addressed the All-Russian Collection of Microorganisms (VKM) for taxonomically related strains (*S. albus*, *S. monomycini* and related *Streptomyces* sp.). The strain *Streptomyces* sp. Ac-502 was positive for the production of antimicrobials. It was cultivated and subjected to solid-phase extraction and activity-guided fractionation. The active component was found to have UV maxima at 214 and 276 nm (Figure 3A). Mass spectrometry revealed an [M+H]^+^ ion with an exact mass of 412.1767 Da, corresponding to the molecular formula C_16_H_25_N_7_O_4_S, which is consistent with a cysteinyl-containing analog of Puro. MS fragmentation (Figure 3C) in the positive mode HCD fragmentation showed predominant fragment ions at *m*/*z* 249.09 and 164.09 due to *N,N*-dimethyladenosine loss. The ion 118.03 was assigned as S-methyl-cysteine moiety. All product ions containing the primary amino group had a characteristic loss of a 17-amu fragment corresponding to the mass of ammonia. For further structure elucidation, the compound was analyzed with NMR.

The NMR data (Figure 3B, Supplement S1) confirmed that the isolated compound is the known antibiotic cystocin (Cst) that was previously identified in *Streptomyces* sp. GCA0001 [24].

### 2.3. Complete Genome Sequencing and Analysis of Cst BGC

**Figure 4:**
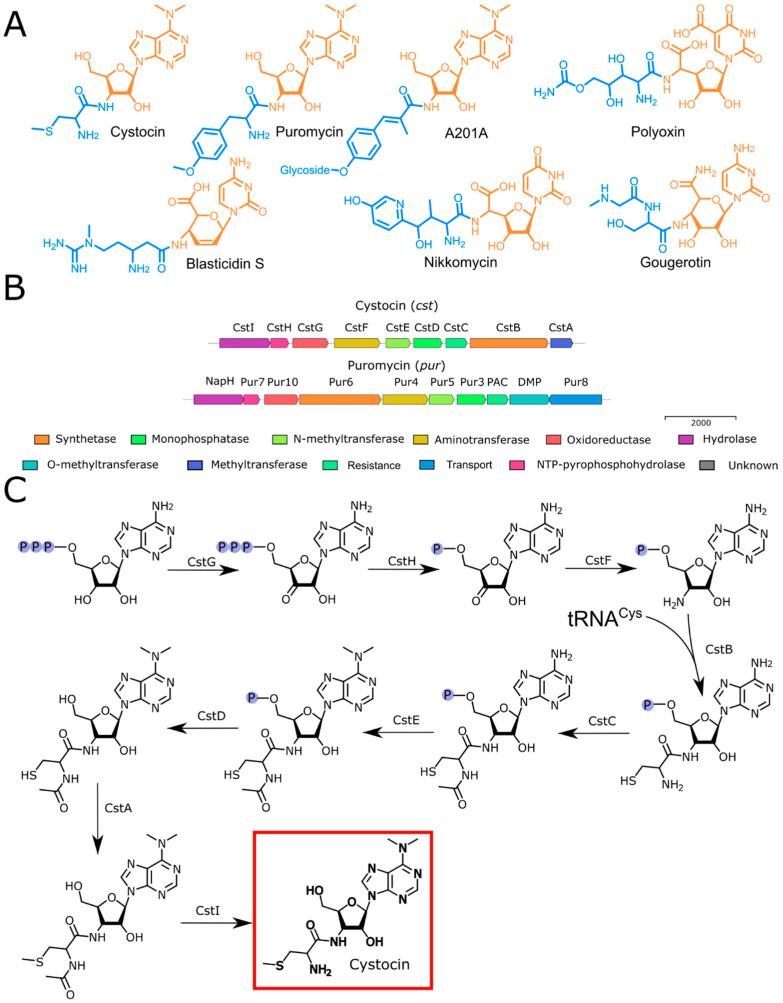
Biosynthesis of aminonucleosides. (**A**) Structures of Cst and related compounds. Nucleoside cores are highlighted in orange, while peptidyl fragments are marked in blue. (**B**) The suggested cst BGC (Orf1-Orf20) with some proposed functions and the comparison with *pur* BGC from *Streptomyces alboniger* strain ATCC 12461 sequence (NZ_CP023695.1). (**C**) The proposed biosynthetic pathway of Cst.

The phylogenetic analysis using the 16S rRNA gene sequence derived from the genome assembly revealed that the Cst-producing strain *Streptomyces* sp. VKM Ac-502 was closely related to *S. monomycini* NRRL B-24309^T^, *S. ochraceiscleroticus* NRRL ISP-5594^T^, and *S. violens* NRRL ISP-5597^T^ (Figure S1). The analysis of the complete genome sequence showed the aminonucleoside BGC, which was named the *cst* BGC. Cst belongs to a diverse group of peptidyl nucleoside antibiotics [25]. The compounds of similar biosynthetic origin contain a nucleoside core and peptidyl moiety (Figure 4A). While the biosynthesis of Puro has been studied in detail [10,11,12,13,14,15,16,17,18,19,20,21], as far as we know, the biosynthesis of Cst has not been reported.

The overall architecture (Figure 4, Supplement S2) of the *cst* cluster and its protein similarity were analyzed and compared primarily with established peptidyl nucleosides, particularly with Puro (Table S1, Figure 4B).

*Cst* BGC contains a set of enzymes (CstH, CstG, CstF, CstE, CstD, CstC), homologs to those involved in the assembly of the nucleoside moiety of Puro in *pur* BGC (Figure 4B). The nucleoside moiety of a similar antibiotic, A201A, is also assembled by the set of homologous enzymes (ataP3/pur3, ataP4/pur4, ataP5/pur5, ataP7/pur7, and ataP10/pur10) [26,27,28,29]. Moreover, a recently isolated antibacterial prodrug mimic of GTP, ADG, possesses a nucleoside nature [30]. Its BGC includes homologs of the *pur7*, *pur10*, *pur3*, and *pur4* genes, which indicates their participation in the formation of the nucleoside core of the molecule.

The enzyme encoded by the *cstG* gene displays a 61% similarity to the product of *pur10*, which participates in the conversion of a hydroxyl group to a carbonyl group during puromycin biosynthesis [11]. A similar activity was observed for the product of the *rifL* gene during rifamycin biosynthesis, which has a high degree of similarity at the amino terminus (with identity 32%) with the product of *pur10* [31,32]. CstG, along with Pur10 [10], functions as an NAD-dependent ATP dehydrogenase.

CstH exhibits a 64% identity with the product of the *pur7* gene in the *pur* BGC. Pur7 is a nudix hydrolase [20] responsible for producing pyrophosphate and the relevant NMP from NTP. CstF is an aminotransferase that is likely involved in the subsequent formation of 3′-amino-3′-dAMP, showing a similarity of 78% to Pur4. The dephosphorylation of the nucleoside core of Puro is performed by the product of the *pur3* gene (with a similarity of 69% to CstD), which is essential for puromycin biosynthesis [21].

Cst and Puro have different types of methylation, S- and O-methylation, respectively. Hence, the *cst* BGC does not have a homolog of the *dmpM* gene, which is required for the O-methylation of tyrosine [17]. The proposed S-methylation of cystocin is mediated by the product of the *cstA* gene—a methyltransferase that exhibits low identity to the O-methyltransferase DmpM.

The attachment of the tyrosine moiety to the nucleoside core of Puro is mediated by Pur6 [11,12]. Within the *cst* BGC, the introduction of Cys is mediated by an aminonucleoside synthase referred to as CstB. CstB has modest similarity (53%) to Pur6, presumably also involving tRNA^Cys^ as an amino acid substrate. This type of aminonucleoside synthase is specific for Puro-like antibiotics. tRNA-dependent peptide bond formation, mediated by the transferase PacB, has been observed in the biosynthesis of pentapeptidyl nucleoside antibiotics pacidamycins [33]. Enzymatic machinery responsible for assembling the nucleoside and peptidyl moieties is different from other peptidyl nucleoside antibiotics. Most of the aminonucleosides are synthesized with ANP-grasp-fold superfamily enzymes (NikS for nikkomycin [34], BlsL for blasticidin S [35], SanS and PolG for nikkomycin analogs [36] and polyoxins [37]). Members of this superfamily possess a unique structure in their ATP-binding sites and catalyze the ATP-dependent coupling of a carboxylic acid with a nucleophile, with the formation of an acylphosphate intermediate [38]. Alternatively, peptidyl moiety can be activated with adenylation (e.g., NpsA in biosynthesis of streptothricins [39]) or CoA-binding (e.g., GouK-activated seryl or sarcosyl groups are transferred by an acyl-CoA N-acyltransferase GouJ from acyl-CoA to the amino group of the gougerotin nucleoside moiety [40]).

Resistance to Puro is mediated by the N-acetyltransferase PAC [14]. Puro is initially produced as an inactive form (N-acetylpuromycin). This compound is actively transported out of the cell, and on the cell’s exterior, the acetamide group is hydrolyzed by the N-acetylhydrolase NAPH, releasing the active Puro into the surrounding environment [15]. Within the *cst* BGC, we have identified an N-acetyltransferase CstC, which shares a significant similarity (66%) with N-acetyltransferase PAC. Hence, we suggest that CstC also inactivates Cst by N-acylation. In turn, CstI mediates the reactivation of Cst since it has a moderate similarity (54%) with NapH [11].

### 2.4. CstC Mediates Resistance to Cst

**Figure 5:**
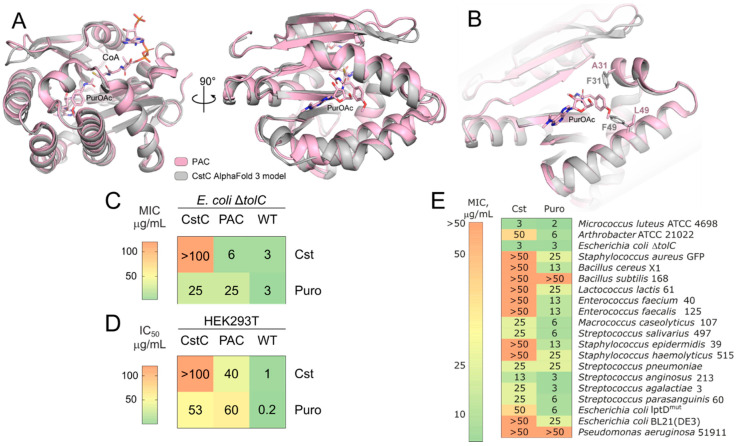
CstC mediates resistance to cystocin. (**A**) Structural alignment of the CstC AlfaFold 3 model (grey) with the PAC-PuroOAc-CoA complex (pink), PDB ID 7K0A. (**B**) Antibiotic-binding pocket of CstC and PAC. The 31 and 49 positions located proximately to the aminoacyl residue of Puro-OAc are highlighted with sticks. (**C**) Comparison of bacterial susceptibility for *E. coli* Δ*tolC* and *E. coli* Δ*tolC* producing CstC or PAC. (**D**) Cytotoxicity of Cst and Puro toward human cell line HEK293T and HEK293T producing CstC or PAC. (**E**) Heat map of MICs of Cst and Puro against Gram-positive and Gram-negative bacteria.

To understand the differences in functionality of aminonucleoside-inactivating N-acetyltransferases, PAC and CstC were studied. The CstC AlfaFold 3 structure model is very similar to PAC, having an RMSD = 0.53 Å. Two amino acid substitutions in the CstC model, A31F and L49F, were located at a distance less than 3 Å and 1 Å to the O-methyltyrosine residue of Puro, resulting in a more bulky active site and steric hindrance for Puro in CstC (Figure 5A,B). Steric hindrance of the antibiotic-binding pocket results in CstC preference for cystocin as a substrate.

Aminonucleosides have potent antibacterial activity against hypersensitive bacterial strains (Figure 5C) and cytotoxicity toward mammalian cells (Figure 5D, Table S2). Recombinant production of CstC in *E. coli* Δ*tolC* mediates its resistance to Cst, increasing MIC values more than 16 folds (Figure 5C). It also provides partial resistance toward Puro. On the contrary, recombinant production of PAC does not influence *E. coli* Δ*tolC* resistance to Cst, making the bacteria resistant to Puro (Figure 5C). Even higher resistance of N-acetyltransferase-producing mammalian cells was observed (Figure 5D). PAC-producing cells were resistant to both Cst and Puro, although the acquired resistances were higher for Puro than Cst. CstC-producing cells, in their turn, acquired higher resistance to Cst (Figure 5C,D).

To assess natural bacterial resistance to Cst and Puro, susceptibility testing against a wide range of Gram-positive and Gram-negative bacteria, including hypersensitive strains of *Escherichia coli lptD^mut^* and Δ*tolC*, was performed (Figure 5E). Cst is active against *Micrococcus luteus* ATCC 4698, *E. coli lptD^mut^*, *E. coli* Δ*tolC*, *Arthrobacter* ATCC 21022, and *Macrococcus caseolyticus* 107. The activity of Puro was 2–8 times higher than that of Cst, and it has a broader activity spectrum. A high activity against *E. coli* Δ*tolC* compared with *E. coli lptD^mut^* suggests that Cst resistance is mediated by efflux in Gram-negative bacteria.

### 2.5. Cst Acts by Translation Inhibition

**Figure 6:**
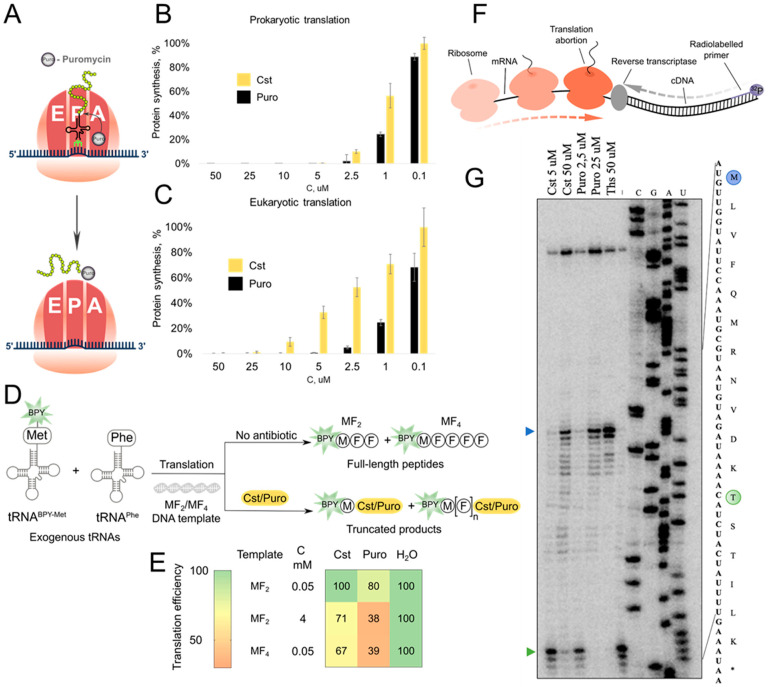
Cst mode of action. (**A**) Schematic representation of Puro mode of action. In vitro prokaryotic (**B**) and eukaryotic (**C**) protein synthesis inhibition by Puro and Cst. (**D**) Fluorescently labeled short peptides synthesis inhibition assay scheme, labeled peptides MF_2_ and MF_4_ are synthesized with coupled transcription–translation from exogenically formed loaded tRNA and visualized using PAGE with fluorescent detection. BPY—BODIPY. (**E**) Heatmap of normalized intensity of full-length peptides under treatment with Cst/Puro. (**F**) The principal scheme of toe-printing analysis. (**G**) Toe-printing analysis for Cst/Puro. The green arrow indicates the position of ribosome capture on the Thr codon; the blue arrow indicates the start codon. Ths—thiostrepton.

Puro inhibits translation by incorporation into the growing peptide chain, leading to the premature release of puromycylated peptides (Figure 6A). The Cst antibacterial mechanism was first assessed using reporter *E. coli* strain JW5503 Δ*tolC* pDualrep2, which consists of two fluorescent protein reporter genes, *turborfp* (induced by SOS-inducible *sulA* gene promoter) and *katushka2S* (regulated by the modified *trpL* attenuator sequence) [41]. Expression of Katushka2S under treatment with a sublethal concentration of Cst suggested protein synthesis inhibition by the isolated compound (Figure S2).

Cst efficiently inhibited protein synthesis in both prokaryotic and eukaryotic cell-free translational systems (Figure 6B,C). Cst has a comparable activity level with Puro in blocking bacterial protein synthesis (0.9 ± 0.4 μM and 0.2 ± 0.1 μM for Cst and Puro, respectively), being 8-fold less active in the eukaryotic system (3 ± 2 μM and 0.4 ± 0.4 μM for Cst and Puro, respectively).

To understand the mechanical details of the inhibition of the peptidyl-transfer reaction, visualization of the synthesized fluorescently labeled peptides is provided (Figure 6D). In line with the previous results, Puro was more active than Cst, inhibiting the synthesis of very small MFF peptides (Supplement S3). For longer MFFFF peptides, Cst inhibition was more efficient, indicating more sufficient synthesis blocking of long templates (Figure 6E). The formation of cystocinilated and puromycinilated truncated peptides was corroborated with LCMS analysis (Supplement S4).

The toe-printing analysis confirmed similar modes of translation inhibition by Cst and Puro (Figure 6F). In high concentrations (Figure 6G), both Puro and Cst inhibited translation at the start codon or near it. However, in the case of Puro, the inhibition occurs completely so that ribosomes do not reach the threonine codon, while for reaction with cystocin, a part of ribosomes reaches the threonine codon and we can detect ribosome capture at the Thr codon caused by borrelidin. Therefore, Cst is more important in blocking the synthesis of large proteins.

## 3. Discussion

Despite the widespread use of puromycin in biotechnology, its biosynthesis is still not described in molecular details. Genome mining based on the key biosynthetic enzyme—unique aminonucleoside synthase Pur6—provides clues for its activity. We propose that Pur6-like enzymes use aminoacyl-tRNAs as a substrate for the amino acid-nucleotide coupling reaction. In this case, *Streptomyces* may utilize their genome-encoded tRNAs. In contrast, *Xenorhabdus* appears to have acquired puromycin BGC through horizontal gene transfer from *Streptomyces* or *Actinomycetia*, such as *N. kunsanensis*, and adapted it by incorporating additional tRNA, as tRNAs in Gram-positive and Gram-negative bacteria are not interchangeable for Pur6-like enzymes.

Analysis of related clusters allowed us to evaluate and access the natural structural diversity of aminonucleoside antibiotics, revealing the *cst* BGC. Distinct BGC architecture and its association with tRNA^Cys^ allowed us to cluster Cst-producing bacteria, including *Streptomyces* sp. VKM Ac-502. In silico analysis of the BGC and structural elucidation of the biosynthetic product both confirmed the production of the cysteinyl-containing Puro analog, i.e., Cst. Notably, there exists a naturally occurring bacterial puromycin-related metabolite with a 3′-N-amino acid substitution differing from the classical 3′-N-tyrosinyl. In the case of puromycin B, a leucine residue replaces the tyrosine. The biosynthesis of such compounds involves a promiscuous aminonucleoside synthetase [42]. Further exploration of the amino-nucleoside synthase could potentially lead to the discovery of novel peptidyl nucleoside antibiotic congeners. Our findings suggest that the directed evolution of Pur6-like enzymes could expand the chemical space of the puromycin antibiotic family by diversifying the peptidyl moieties.

The biosynthesis of aminonucleoside antibiotics includes specific self-resistance mechanisms, such as N-acetylation of the produced antibiotic, rendering it inactive within the producing organism. Puro-based systems for the selection of cell lines utilize this resistance mechanism. The development of modified variants of PAC for improved selection systems still attracts significant attention [43]. The *cst* BGC contains an N-acetyltransferase, CstC, with a similar function. The specificity of CstC is different from that of the PAC enzyme. Susceptibility testing revealed that both CstC and PAC provide some level of cross-resistance. However, the acquired resistance is significantly higher for the corresponding aminonucleoside. This effect is mediated by the bulky substituents in close proximity to the catalytic center of CstC, making it less spatially accessible for Puro. This finding could provide a basis for the rational design of selective resistance enzymes to develop selection systems orthogonal to puromycin-based ones.

Puro is a well-known translation inhibitor and is widely used in scientific research. Cst is similar to Puro in many ways: it is toxic to Gram-positive bacteria, suppresses protein biosynthesis in vitro in cell-free translation systems, and at high concentrations, even inhibits the biosynthesis of short peptides. Despite structural homology to tyrosyl-tRNA, Puro incorporation is known to be not amino acid specific [5]; the same mode of action was observed for Cst. Analyzing the toe-prints and small peptides labeled with BODIPY, we came to the conclusion that the effect of both puromycin and cystocin is cumulative. That is why the longer the template, the more frequently the transfer occurs. On short templates, the inhibition effect is noticeable only at very high concentrations, when aminonucleoside saturates all ribosomes. The obtained results generalize the diversity of aminonucleoside synthetases, providing a basis for the further discovery of new aminonucleosides. Moreover, the discovered mechanistic details could be applied to expand the biosynthetic potential of Pur6-like enzymes and provide selective aminonucleoside-inactivating enzymes.

The findings presented in this manuscript highlight the significant biotechnological potential of aminonucleoside-based systems for further development in molecular biology. However, the currently obtained results come with certain limitations, which hinder their immediate practical application. The first studied enzyme, aminonucleoside synthase, demonstrates the potential for directed evolution to produce novel aminonucleoside antibiotics with unique biological functions. Additionally, this study successfully obtained only one strain from the cst-like BGC-bearing clade, suggesting that further mining of the strains related to the identified *Streptomyces* and *Xenorhabdus* species could uncover new natural congeners with potentially distinct properties.

Moreover, the self-resistance enzyme CstC shows promise as a candidate for the development of an orthogonal selective marker in molecular biology. However, the current Cst/Puro selectivity parameters require further optimization. The identified amino acid substitutions, A31F and L49F, which introduce steric hindrance in the active site, represent promising starting points for the engineering of highly selective N-acetyltransferases. Future studies focusing on enzyme optimization and exploring the diversity of aminonucleoside-producing strains could unlock new avenues for the practical application of these systems in biotechnology.

## 4. Materials and Methods

### 4.1. Genome Sequencing and Data Analysis

Pur6 homologs were identified among non-redundant protein sequences using protein BLAST [44]. Pur6-like enzymes had E-value > 1 × 10^−40^, cover > 76%, protein identity > 28%, similarity > 40%. Pur6-like enzymes were downloaded from GenBank with adjacent 100 kb genome fragments and processed with antiSMASH to identify BGCs [45]. A tree of Pur6-like enzymes was built with MAFFT [46]. BGC analysis was performed with CAGECAT [47]. tRNAs were detected with tRNAscan-SE 2.0 [48], and alignments were visualized with MView [49]. Structure models were built with AlphaFold 3 [50]. Protein sequences with the following accession numbers were used for phylogenetic tree construction: WP_026116056.1, WP_074025210.1, WP_021324692.1, WP_099109202.1, WP_010848540.1, WP_041976841.1, WP_013185625.1, CEE90880.1, WP_339351399.1, SFU95741.1, WP_245759091.1, WP_258087068.1, WP_030021787.1, WP_125058165.1, WP_030888603.1, WP_030885422.1, WP_301126629.1, WP_086817987.1, WP_149564235.1, WP_159789347.1, WP_030778222.1, WP_055528327.1, WP_069885355.1, WP_055700141.1, WP_093842894.1, WP_176127489.1, WP_019433835.1, WP_031508068.1, WP_184730161.1.

The DNA was isolated using the Wizard DNA extraction kit (Promega Corporation, Madison, WI, USA) and size-selected with optimized solid-phase reversible immobilization (SPRI) beads. The DNA concentration and quality were determined on a Qubit 4 Fluorometer and Nanodrop ND-1000 (Thermo Fisher Scientific, Waltham, MA, USA). The long reads were generated with MinION sequencing (Oxford Nanopore Technologies, Oxford, UK). The sequencing libraries were prepared using the ligation sequencing kit SQK-LSK109 and native barcoding expansion kit EXP-NBD114 and run in a FLO-MIN106 flow cell. Reads were basecalled using Guppy v3.6.1. with default parameters (guppy_basecaller). A NEBNext Ultra DNA library prep kit (New England Biolabs, Ipswich, MA, USA) was used to prepare fragment libraries for genome sequencing. Sequencing was performed on the HiSeq 2500 system (Illumina, San Diego, CA, USA) HiSeq SBS Kit v4 (250 Cycle) using a 2 × 125 bp run configuration. De novo assembly was performed by Flye assembler (2.8.1) [51] using default parameters. Illumina reads were used to improve and correct genome assembly using the Pilon program [52]. Identification of the protein-coding sequences and primary annotation were performed using PROKKA v1.14.6 [53]. The draft genome sequences and raw sequencing reads have been deposited at GenBank, BioProject accession number PRJNA1158227, GeneBank accession number JBHFFO000000000.

The genome of *Streptomyces* sp. VKM Ac-502 was analyzed with antiSMASH to identify potential BGCs [45]. Homologous gene clusters were identified with MultiGeneBlast [54] using the MIBiG database [55]. Putative functions of enzymes were assigned according to the predicted function of the closest characterized relative identified by Blast search [44] in NCBI.

A truncated puromycin BGC sequence was acquired from the MiBIG database (BGC0000878). Complete puromycin BGC was acquired from *Streptomyces alboniger* ATCC 12461 sequence (NZ_CP023695.1). Additional tailoring enzyme sequences were acquired from Genbank: *pur8* (X76855.1), *dmpM* (M74560.2), and *pac* (M25346.1).

The generic affiliation of *Streptomyces* sp. VKM Ac-502 was initially determined on the basis of a 16S rRNA sequence derived from the genome assembly of strain. The 16S rRNA gene sequence (1451 bp) similarities between the target strain and the type strains of known *Streptomyces* species were estimated using the EzBioCloud server (https://www.ezbiocloud.net (accessed on 20 August 2024) [56]). A digital DNA-DNA hybridization (dDDH) [57] and Average Nucleotide Identity (ANI) values [58,59,60] between VKM Ac-502 and type strains of *Streptomyces* species were calculated using the Type (Strain) Genome Server (https://tygs.dsmz.de/user_requests/new (accessed on 20 August 2024) [61]) and JSpecies WS tool (https://jspecies.ribohost.com/jspeciesws (accessed on 20 August 2024) [62]), respectively.

### 4.2. Bacterial Strains and Cell Lines

The antibacterial activity of the strains was evaluated using a hypersensitive *E. coli* Δ*tolC* strain transformed with pDualrep2 reporter plasmid [41]. The cystocin-producing strain *Streptomyces* sp. VKM Ac-502, also known under the invalid species name “*Streptomyces tumemacerans*” [63,64], was obtained from the All-Russian Collection of Microorganisms (www.vkm.ru).

A bacterial collection including *Micrococcus luteus* ATCC 4698, *Bacillus cereus* X1, *Lactococcus lactis* 61, *Enterococcus faecium* 40, *Enterococcus faecalis* 125, *Macrococcus caseolyticus* 107, *Staphylococcus epidermidis* 39, *Staphylococcus haemolyticus* 515, *Pseudomonas aeruginosa* 51911, *Streptococcus pneumonia* ATCC 6303, *Streptococcus anginosus* 213, *Streptococcus agalactiae* 3, *Streptococcus parasanguinis* 60, and *Streptococcus salivarius* 497 was kindly provided by Lytech Co., Ltd. (Moscow, Russia) [65]. Staphylococcus aureus GFP constitutively producing GFP was kindly provided by Andrey Shkoporov from the Department of Microbiology and Virology, Russian National Research Medical University, Moscow. The *E. coli* ΔtolC KanS, *E. coli* lptD^mut^, and *E. coli* strain JW5503 (ΔtolC) [66] transformed with pDualrep2 reporter plasmid [41] were used from our laboratory strain collection. Strains *Arthrobacter* sp. ATCC 21022, *Bacillus subtilis* 168, and *Escherichia coli* BL21(DE3) were purchased from Invitrogen Corp. (Carlsbad, CA, USA). Cell line HEK293T cells were kindly provided by Dr. E. Knyazhanskaya.

### 4.3. Cultivation, Isolation, and Purification of Cystocin

The strain *Streptomyces* sp. VKM Ac-502 was transferred from the surface of the agar medium into a 750 mL Erlenmeyer flask with 50 mL of LB nutrient medium of the following composition (components content in g/L): tryptone—10, yeast extract—5, NaCl—10, distilled water—up to 1 L, pH 7.4. Cultivation was carried out at 28 °C for 4 days on a thermostat shaker Innova 40 (New Brunswick Scientific, Middleton, WI, USA) at 150 rpm. The seed culture of the second generation was grown in 150 mL of LB medium under similar conditions for 6 days, using the first inoculum in an amount of 3% vol. The strain produced cystocin starting from 2 days of cultivation and reached its maximum production on day 6 of cultivation.

Bacterial cells were eliminated from culture broth by centrifugation at 5000 rpm on a Sigma 3–16KL centrifuge and filtration through a 0.47 μm MCE membrane filter (Millipore, Billerica, MA, USA). A total of 1 L of clarified supernatant was loaded on the 30 mL cartridge packed with 7 g of LPS-500-H polymer sorbent (copolymer divinylbenzene—hydrophilic monomer, pore size 50–1000 Å, 70 μm, Technosorbent, RF) at flow rate 15 mL/min using a peristaltic pump (Masterflex L/S variable speed pump systems, Masterflex, Gelsenkirchen, Germany). Extraction by a water/acetonitrile (MeCN) mixture was performed at a flow rate of 15 mL/min in gradient mode from 0 to 100% ACN for 10 min using a PuriFlash 5.250 instrument (Interchim, Montluçon, France), and 15 mL fractions were collected. The activity of the collected fractions was tested using the reporter *E. coli* Δ*tolC* pDualrep2. The most active fraction (eluted at about 20% MeCN) was further analyzed using HPLC on an RP column using a Nexera X2 LC 30A instrument (Shimadzu, Kyoto, Japan) equipped with an SPD-M20A detector (Shimadzu, Kyoto, Japan). HPLC conditions were as follows: column Zorbax SB C18 4.6 × 150 mm, 5 μm, eluent solvent A—10 mM NH_4_OAc, pH 4.5, solvent B—MeCN; gradient elution from 5 to 90% of solvent B; flow rate 1.5 mL/min; UV detection at 275 nm. HPLC fractions were collected and tested for activity, and the fraction containing pure active substance (Figure S3) was isolated and then analyzed by LCMS. Isolation of the active compound for NMR experiments and biological assays was performed using semipreparative column Zorbax SB C18 9.4 × 150 mm, 5 μm, with solvents A and B as above at 16% of solvent B in isocratic mode and at the flow rate 2.5 mL/min.

### 4.4. LCMS Analysis

LC-MS analysis was carried out on an Ultimate 3000 RSLCnano HPLC system connected to an Orbitrap Fusion Lumos mass spectrometer (ThermoFisher Scientific, Waltham, MA, USA) with the loading pump used for analytical flow gradient delivery. Samples were separated on a Gemini NX-C18 3 µm 100 Å column 100*2.1 mm at 200 µL/min flow rate in the linear gradient of acetonitrile in water with the addition of 10 mM ammonium formate and 0.1% FA. UV data were collected at 275 nm. MS1 and MS2 spectra were recorded at 30 K and 15 K resolution, respectively, with HCD fragmentation. Raw data were collected and processed on Thermo Xcalibur Qual ver. 4.3.73.11.

### 4.5. Structure Elucidation

The structure of cystocin was elucidated using the conventional heteronuclear NMR approach. A total of 1.5 mg of pure compound was dissolved in 350 uL DMSO-d_6_ (99.98% purity, CIL, Tewksbury, MA, USA) and placed into the Shigemi DMS-005TB NMR tube (Shigemi, Kawaguchi, Japan). NMR spectra were recorded at 35 and 45 °C using the Bruker AvanceIII 800 MHz NMR spectrometer (Bruker, Billerica, MA, USA) equipped with a TCI cryogenic probe. The 1D ^1^H,^13^C and 2D ^1^H,^13^C-HSQC; ^1^H,^15^N-HSQC; ^1^H,^13^C-HMBC (optimized for 8 Hz J-coupling); ^1^H,^15^N-HMBC (optimized for the 5 Hz J-coupling), DQF-COSY, ^1^H,^13^C-HSQC-TOCSY (20 ms mixing) and ROESY (200 ms mixing) spectra were recorded. The physico-chemical properties of the compound were identical to previously reported [24].

### 4.6. Plate Test Against Escherichia coli Reporter Cells

The *E. coli* strains (JW5503 Δ*tolC* and *lptD^mut^*) transformed with pDualrep2 reporter plasmid were used to evaluate the mechanism of antimicrobial action and activity against bacteria. The overnight culture of the reporter strains was diluted with fresh LB medium to an optical density of 600 nm (OD600) of 0.05–0.1. The cultures were transferred to LB agar plates with 100 μg/mL ampicillin or 50 μg/mL kanamycin applied. On an agar plate with the lawn of the reporter strains, 1 μL of compounds Cystocin (Cst) and Puromycin (Puro) at a concentration of 20 mg/mL were spotted. Erythromycin (Ery, 5 mg/mL) and Ciprofloxacin (Cip, 10 μg/mL) were used as control antibiotics on an agar plate. ChemiDoc (Bio-Rad, Hercules, CA, USA) was used to scan agar plates in the Cy3 (TurboRFP) and Cy5 (Katushka2S) channels after overnight incubation at 37 °C. This was performed to determine the inhibition zone and fluorescence levels of the reporter proteins.

### 4.7. Antibacterial Activity Assessment

Determination of the activity spectra of Cst and Puro for comparative analysis was carried out using the method of serial dilutions. Individual colonies of bacteria from the plate with 2YT agar medium (10 g/L yeast extract, 16 g/L tryptone, 5 g/L NaCl, 15 g/L agar) were transferred to 5 mL of 2YT nutrient medium (10 g/L yeast extract, 16 g/L tryptone, 5 g/L NaCl) and incubated at 37 °C overnight. The resulting overnight cultures were transferred to fresh nutrient medium in a ratio of 1:100 and incubated at 37 °C for 3 h. Next, the cultures were diluted so that the optical density of the final solution at 600 nm was ~0.001 a.u. This cell culture was used to study the spectrum of antibiotic activity (Cst and Puro). Antibiotic MICs were determined after 16 h of incubation in a 96-well plate at a wavelength of 600 nm using a Varioskan multimodal reader (Thermo Fisher Scientific, USA); 50 μg/mL was chosen as the initial concentration for antibiotics with subsequent 2X-fold serial dilutions.

### 4.8. Cytotoxicity Studies

To prepare the MTT solution, a dry sample of thiazolyl blue tetrazolium bromide was dissolved in phosphate-buffered saline, pH = 7.4 (DPBS), to a concentration of 5 mg/mL. The resulting solution was sterilely filtered through a filter with a pore diameter of 0.2 μm, and the filtrate was collected in a sterile container protected from light. To prepare a solubilizing solution, 40% (vol.) dimethylformamide (DMF) was diluted in 2% (vol.) glacial acetic acid. A total of 16% (*w*/*v*) sodium dodecyl sulfate (SDS) was added to the resulting solution. Using 2% (vol.) glacial acetic acid, the pH of the solution was adjusted to 4.7.

Double dilutions of the test compounds were prepared in DMSO as a negative control in the culture medium. A final concentration series of eight 2-fold dilutions was started at 6.4 μg/mL. Incubation was carried out in a humidified atmosphere at 37 °C with 8% CO_2_ for 72 h. Cytotoxicity was assessed in HEK293T cells [67]. Cell viability was determined by colorimetric assessment of cell metabolic activity (MTT). The analysis was carried out after incubation for 72 h. All measurements were performed in triplicate biological replicates. A total of 10 μL per well of MTT solution was added to the test samples to a final concentration of 0.45 mg/mL and incubated for an hour at 37 °C. Then, 100 μL of solubilizing solution was added to each well to dissolve the formazan crystals. The result was measured using a Varioskan multimodal reader (Thermo Scientific, USA) at an absorbance of 570 nm. The obtained results were analyzed using MS Excel 2013 software. Based on the results of the MTT test, the graph of the dependence of the average survival value on the concentration of the test substance plotted on a logarithmic scale was constructed. A well with cells that did not contain the test compounds was taken as 100% viability. Relative survival was calculated using the following formula: (absorption for a given well)/(absorption for a well without adding the drug)·100%. The cytotoxic concentration for each compound (IC_50_) was determined from the resulting inverse sigmoid as the value of the concentration at which the cytotoxic effect is induced in 50% of the cells in the monolayer. Doxorubicin was used as positive control.

### 4.9. Resistance Studies

**Construction of plasmids pSol_CstC and pSolPAC.** The *Streptomyces* sp. VKM Ac-502 genomic DNA was used as a template for obtaining *cstC* sequence flanked with restriction sites for cloning. Primers for PCR were used as F_NdeI_CstC 5′ GCCAATCATATGACCTCGAACGCCCCCG 3′ and R_NotI_CstC 5′ TGACTGCGGCCGCTCAGGCGCCCGGCTTACGG 3′.

The gene of puromycin N-acetyltransferase was amplified from plasmid pCDF1-MCS2-EF1-Puro (System Biosciences, Mountain View, CA, USA) with primers F_NdeI_PAC 5′ GCCtcaCATATGACCGAGTACAAGCCCACG 3′ and R_NotI_PAC 5′ TGACTGCGGCCGCTCAGGCACCGGGCTTGCG 3′ flanked with restriction sites for cloning.

PCR products and vector pSolSumo (Lucigen, Middleton, WI, USA) were digested with restriction enzymes NdeI and NotI (New England Biolabs, Cambridge, UK), followed by ligation with T4 DNA ligase (Evrogen, Moscow, Russia) according to the manufacturer’s protocol. The resulting plasmids pSol_CstC and pSol_PAC were transformed into *E. coli* DH10β and verified by sequencing. The *E. coli* Δ*tolC* kanS cells were transformed with the corresponding plasmids, and the obtained strains were used in antibiotic susceptibility experiments. In pSolSumo, vector gene expression is under the control of rhamnose-dependent rhaB promotor; therefore, different concentrations of rhamnose were used to control the CstC expression rate. However, no significant differences were observed.

**Transformant antibiotic susceptibility testing.** The minimal inhibitory concentrations (MIC) were estimated with a microwell broth dilution assay. In brief, the tested bacteria were diluted in 2YT media (10 g/L yeast extract, 16 g/L tryptone, 5 g/L NaCl) to a final concentration of approximately 1 × 10^6^ CFU/mL in a 96-microwell plate, untransformed *E. coli* Δ*tolC* kanS were used as control. The cystocin and puromycin samples were used to form 2-fold serial dilutions starting with 100 μg/mL. Plates were incubated at 37 °C with shaking at 500 rpm for 16 h. After incubation, the optical density at 600 nm of each well was measured with a Varioskan Flash multimode reader (Thermo Fisher Scientific, USA). MICs were determined as the lowest antibiotic concentration that inhibited the growth of the bacteria. For expression induction, several concentrations of rhamnose were used (from 0 to 1 mM).

**Mammalian cell growth and *pac* and *cstC* resistance genes transfection.** The HEK293T cell line was cultured in a 6-well plate at 37 °C in 5% CO_2_ in DMEM/F12 medium supplemented with 10% fetal bovine serum and Gibco GlutaMAX. Then, two pSBbi-NEO (AddGene 60525) vectors with the corresponding resistance genes were cloned by SfiI restriction followed by sticky ends insertion of the resistance genes obtained with PCR. The puromycin resistance gene, puromycin N-acetyltransferase (*pac*), was amplified from the Human CRISPR KO Library in lentiGuide-Puro (AddGene 1000000049). The cystocin resistance gene, *cstC*, was amplified from the genomic DNA of the producing strain *Streptomyces* sp. VKM Ac-502. Obtained vectors and transposase caring plasmid pCMV (CAT) T7-SB100 (AddGene 34879) were transfected into HEK293 cells using Lipofectamine 3000 (#L3000008, Thermo Fisher Scientific, USA) in accordance with the manufacturer’s protocols, harvested at 24 h and treated with antibiotics at a concentration 5 times higher than the IC_50_. Cell selection with incorporated resistance genes lasted 12 days, then cryostocks were prepared. Afterward, they were applied for further experiments.

### 4.10. In Vitro Studies

**Bacterial in vitro translation assay.** The PURExpress system (NEB) was used to conduct prokaryotic in vitro translation reactions. Each reaction was 5 µL in total, supplied with 0.1 mM of d-luciferin (Promega), 0.5 μL of either antibiotic solution or solvent (water), as indicated, and 100 ng of Fluc mRNA (the latter was added in 1 μL water solution after the reaction mixture supplemented with antibiotic was pre-incubated for 5 min at room temperature). After the mRNA addition, mixtures were transferred into a preheated white FB/NB 384-well plate (Grenier no. 781904) and incubated in the VictorX5 multi-reader at 30 °C with continuous measurement of luciferase activity.

**Mammalian cell-free system.** For testing compounds in a mammalian in vitro translation system, whole homemade HEK293T cell extracts were used. The total volume of the translation reaction was 10 µL, including 5 µL HEK293T extract, 1 µL 10× translation buffer (20 mM Hepes-KOH pH 7.6, 1 mM DTT, 0.5 mM spermidine-HCl, 0.8 mM Mg(OAc)_2_, 8 mM creatine phosphate, 1 mM ATP, 0.2 mM GTP, 120 mM KOAc, and 25 μM of each amino acid), 2U of RiboLock RNase inhibitor (Thermo Scientific, USA), 0.5 mM d-luciferin (Promega), 1 μL of either antibiotic solution or solvent (water), as indicated, and 50 ng mRNA (the latter was added in 1 μL water solution after the reaction mixture supplemented with antibiotic was pre-incubated for 5 min at 30 °C). After adding the mRNA, the mixtures were transferred to a preheated white FB/NB 384-well plate (Grenier no. 781904) and incubated in the VictorX5 multi-reader at 37 °C with continuous measurement of luciferase activity.

Fluorescently labeled short peptides [68]. DNA templates containing short open reading frames coding M, MF, MF2, and MF4 short peptides were synthesized by PCR using the plasmid puC19 with corresponding inserts as a template for amplification. Each template harbored the T7 promoter and the SD sequence.

Coupled transcription–translation was set up in 10 µL reactions using a PURExpress Δ (aa, tRNA) Kit (NEB), with the addition of 20 ng of DNA template (M, MF, MF_2_, and MF_4_). Also, 0.1 µM BPY-Met-tRNA^fMet^, 0.45 µM fMet-tRNA^fMet^, 1 µM Phe-tRNA^Phe^, 100 µM Phe, and 1 μL of either antibiotic solution or solvent (water), as indicated, were added to each reaction. The reactions were incubated for 30 min at 37 °C and divided into two tubes. To one part, 2 µL of 1 M NaHCO_3_ was added, subsequently followed by incubation at 37 °C for 20 min. Reactions were stopped by adding formamide dye (95% formamide, 0.025% [w/v] bromophenol blue, 0.5 mM EDTA). Samples were then preheated for 3 min at 80 °C and loaded to a 10% denaturing PAGE (19:1 AA:bisAA; 1× TBE buffer; 8M urea). Gels were scanned by a Typhoon FLA 9500 Biomolecular Imager (GE Healthcare, Chicago, IL, USA) in the FAM channel with an excitation peak (493 nm) and emission peak (517 nm).

**Toe-print analysis.** The toe-printing assay was conducted according to the protocol described by Orelle et al. [69]. At the first stage, the primers were labeled with [γ-32P] ATP polynucleotide kinase (ThermoFisher, USA) according to the manufacturer’s protocol. Next, in vitro translation of the short-model mRNA was performed using a PURExpress^®^ In VitroProtein Synthesis Kit (New England Biolabs, USA). The reaction mixture (volume, 5 μL) contained 2 μL of solution A, 1 μL of solution B, 0.2 μL of RiboLock (ThermoFisher, USA), 0.5 μL of the test compound, 0.5 μL of DNA template (0.2 mmol/μL), and 0.5 μL of the radiolabeled primer. A total of 50 μM of borrelidin was added to each sample to stop the translation on the threonine codon.

The mixture was incubated at 37 °C for 20 min, and 1 μL of the reverse transcription mix from the Titan One Tube RT-PCR System kit (Roche, Basel, Switzerland) was added. Reverse transcription was conducted for 15 min at 37 °C. The reaction was stopped by adding 1 μL of 10 M NaOH, followed by incubation at 37 °C for 15 min. The neutralization was performed by adding 1 μL of 10 N HCl. Next, 200 μL of the resuspension buffer was added. The resulting samples were purified using a QIAquick PCR purification kit (Qiagen, Hilden, Germany). The sequence mixtures were prepared using a USB^®^ Thermo Sequenase Cycle Sequencing Kit (Affymetrix, Santa Clara, CA, USA) according to the manufacturer’s protocol. Electrophoresis was carried out in 6% polyacrylamide gel (60 × 40 × 0.03 cm) containing 19% acrylamide, 1% N,N′-methylenebisacrylamide, and 7 M urea in TBE buffer for 2–3 h.

The specimens and products of the sequencing reactions (2 and 1.5 μL, respectively) were applied to the gel. The gel was transferred onto 3-mm paper, dried, and exposed to a sensory screen for 18 h. The screen was scanned using a Typhoon FLA 9500 Biomolecular Imager (GE Healthcare, Chicago, IL, USA). The ermCL template for this experiment was obtained by PCR amplification using a Taq-DNA-polymerase kit (ThermoFisher, USA), according to the standard protocol. The template sequence is described in Table S3.

## Data Availability

The data presented in this study are openly available in NCBI at accession number JBHFFO000000000.

## Supplementary Materials

The following supporting information can be downloaded at https://www.mdpi.com/article/10.3390/ijms252312901/s1.
