# Supplementary material for: Mining Translation Inhibitors by a Unique Peptidyl-Aminonucleoside Synthetase Reveals Cystocin Biosynthesis and Self-Resistance
Source: Int J Mol Sci. 2024 Nov 30;25(23):12901. doi: 10.3390/ijms252312901 (PMC11641026; doi:10.3390/ijms252312901)
Supplement: Supplementary file 1 [file ijms-25-12901-s001.zip › ijms-3331172-supplementary.pdf]

Mining translation inhibitors by a unique peptidyl-aminonucleoside synthetase reveals cystocin biosynthesis and self-resistance

Vera A. Alferova[1†], Polina A. Zotova[2†], Anna A. Baranova[1], Elena B. Guglya[1], Olga A. Belozerova[1], Sofiya O. Pipiya,[1] Arsen M. Kudzhaev,[1] Stepan E. Logunov,[1] Yuri A. Prokopenko[1], Elisaveta A. Marenkova,[1] Valeriya I. Marina[2], Evgenia A. Novikova[2], Ekaterina S. Komarova[3], Irina P. Starodumova,[1,4] Olga V. Bueva[4], Lyudmila I. Evtushenko[4], Elena V. Ariskina[4], Sergey I. Kovalchuk[1], Konstantin S. Mineev,[1] Vladislav V. Babenko[5], Petr V. Sergiev[2,3,6], Dmitrii A. Lukianov[2,6], Stanislav S. Terekhov[1,*]

[1] Shemyakin-Ovchinnikov Institute of Bioorganic Chemistry, Miklukho-Maklaya 16/10, Moscow 117997, Russia

[2] Department of Chemistry, Lomonosov Moscow State University, Moscow 119992, Russia

[3] A.N. Belozersky Institute of Physico-Chemical Biology, Lomonosov Moscow State University, 119991, Moscow, Russia.

[4] All-Russian Collection of Microorganisms (VKM), Pushchino Scientific Center for Biological Research, Russian Academy of Sciences, Pushchino, 142290, Russian Federation.

[5] Lopukhin Federal Research and Clinical Center of Physical-Chemical Medicine, Malaya Pirogovskaya Str. 1a, Moscow, 119435 Russia

[6] Center for Molecular and Cellular Biology, Moscow, Skolkovo, 121205, Russia

[†] These authors contributed equally to this work.

Corresponding Author: Stanislav S. Terekhov, sterekhoff@gmail.com

Supplementary materials

Contents

Supplement S1. Detailed discussion of Cst structure elucidation 3

Figure S1. Phylogenetic analysis of the strain VKM Ac-502 5

Table S1. Cystocin biosynthetic gene cluster (*cst* BGC) analysis.. 7

Supplement S2. The expanded analysis of the *cst* BGC 9

Table S2. Susceptibility of WT. CstC and PAC-transformed eukaryotic HEK293T WT .. 11

Figure S2. Reporter strain screening of Cst and Puro 12

Supplement S3. BODIPY-labelled small peptides synthesis inhibition experiment........... 13

Supplement S4. MS analysis of small peptides synthesis inhibition reactions 17

Figure S3. HPLC-trace of the isolated cystocin fraction.. 19

Table S3. Sequences of templates and primers used for toeprinting assay 20

Supplemental references... 21

Supplement S1. Detailed discussion of Cst structure elucidation

According to the NMR analysis, the compound comprises a purine aromatic system with two characteristic singlets at 8.44 and 8.29 ppm. These singlets provide the connectivities to three quaternary carbons and four ternary nitrogens of purine in HMBC, indicating that the purine is substituted at positions 6 and 9. While substituent at position 6 was not observed in HMBC, it was clear that position 9 was substituted by a five-membered sugar ring, with the 9-1' connection, typical for nucleosides (Fig. 3B). In turn, the 3' position in the sugar is occupied by the nitrogen atom, bound to the aminoacid carboxyl group via the peptide bond. This amide group provides the only peak that can be observed in $^{1}H,^{15}N$-HSQC (8.24; 108.7 ppm). The aminoacid could be identified as S-methylcysteine, the amino-group is observed in HMBC, while the presence of sulfur can be assumed based on the typical chemical shift values. The HRMS data ($[M+H]^+$: 412.1767 Da) allows identifying the last unknown - the purine substituent at position 6, which appears to be N-dimethyl group. The latter was at the end found in ^{1}H spectrum as a broad singleton at 3.45 ppm, which becomes narrower upon heating, apparently due to the hindered rotation relative to the purine ring.

Back to Contents

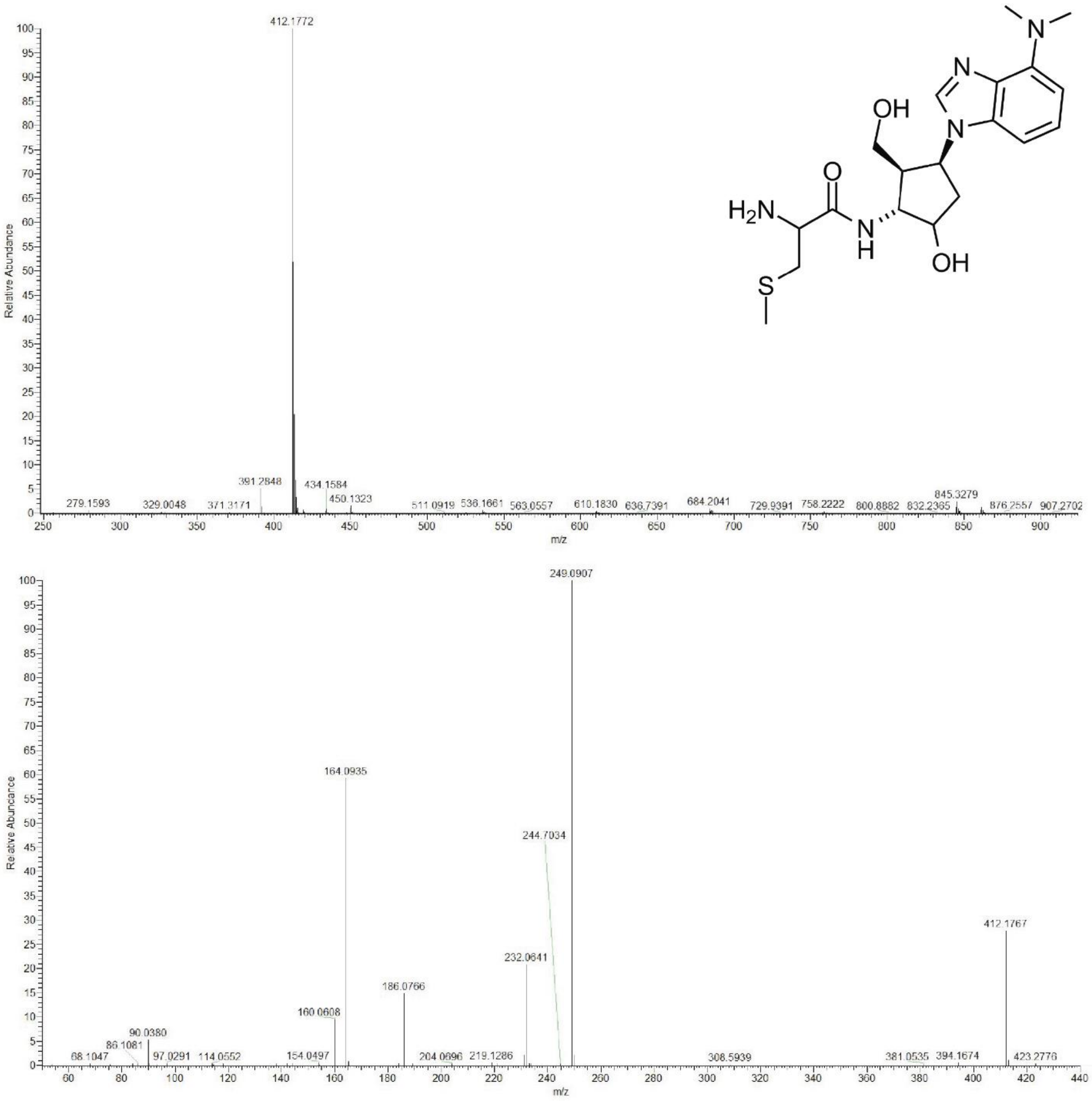

Figure S1-1. MS1 and MS2 data collected at positive mode for m/z 412.1772 (Cst).

Back to Contents

Figure S1. Phylogenetic analysis of the strain VKM Ac-502

Based on 16S rRNA gene sequence comparison, the strain was supported to belong to the genus *Streptomyces*, exhibiting the highest sequence similarity to *S. monomycini* NRRL B-24309^{T} (99.87%), *S. ochraceiscleroticus* NRRL ISP-5594^{T} (99.45%), and *S. violens* NRRL ISP-5597^{T} (99.45%) (Figure S2). The calculated dDDH value between VKM Ac-502 and *S. monomycini* NRRL B-24309^{T} (67.0%) and between VKM Ac-502 and the type strains of other *Streptomyces* species (<49%) were lower than the recommended cut-off value (70%) for distinguishing prokaryotic species [1,2]. Similarly, the ANI values (ANIb, 94.9% and ANIm, 96.3%) determined for the pair VKM Ac-502 and *S. monomycini* NRRL B-24309^{T} were below the thresholds for species delineation (95–96% and 96.7%, respectively) [2–4]. The ANI values for VKM Ac-502 with respect to other *Streptomyces* species were significantly lower than the figures given above. Thus, the species *Streptomyces* sp. VKM Ac-502, originally described as “*Actinomyces tumemacerans*” [5] and “*Streptomyces tumemacerans*” [6] differs from all known species with validly published names and calls for its valid re-description.

Back to Contents

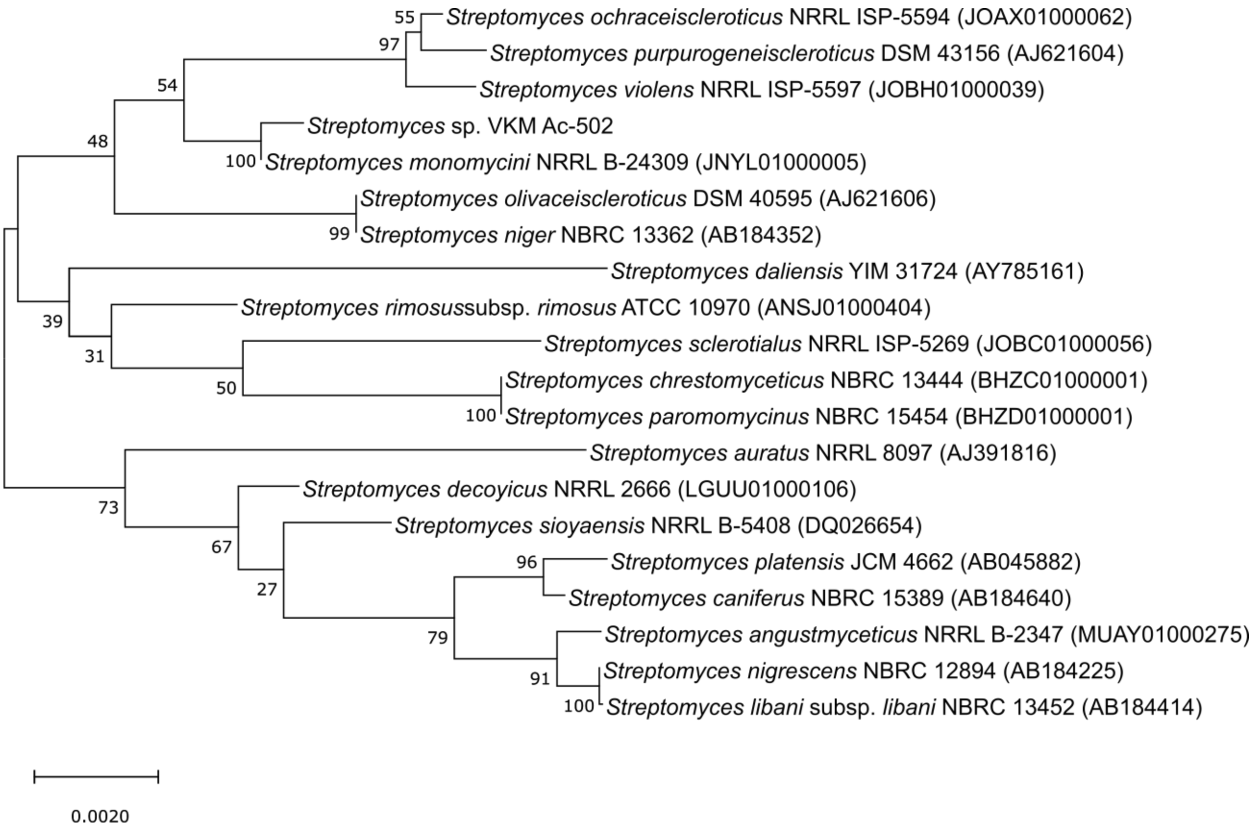

Neighbor-joining phylogenetic tree based on 16S rRNA gene sequence analysis showing the position of strain *Streptomyces* sp. VKM Ac-502 and related species. Bootstrap values based on 1000 resampled datasets are given for values higher than 60%.

Back to Contents

Table S1. Cystocin biosynthetic gene cluster (*cst* BGC) analysis

Orf	Gene name	Gene size (bp)	Proposed function	Protein [Organism]. Corresponding to Gene with Sequence Similarity	NCBI Gene Bank Accession Number	Puromycin BGC homologue	E-value	Protein identity
1	Orf1	1572		AMP-binding protein [Streptomyces monomycini]	WP_030021793.1	-	-	-
2	Orf2	306		putative quinol monooxygenase [Streptomyces monomycini]	WP_030021792.1	-	-	-
3	Orf3	132		hypothetical protein [unclassified Streptomyces]	WP_215157344.1	-	-	-
4	Orf4	918		hypothetical protein [Streptomyces albofaciens]	WP_150249738.1	-	-	-
5	Orf5	513		nuclear transport factor 2 family protein [Streptomyces sp. WM6378]	WP_053729042.1	-	-	-
6	Orf6	855		helix-turn-helix domain-containing protein [Streptomyces chrestomyceticus]	WP_125043395.1	-	-	-
7	Orf7	444		hypothetical protein [Streptomyces sp. MST-110588]	WP_242584962.1	-	-	-
8	Orf8	978		No significant similarity found		-	-	-
9	Orf9	405		ATP-binding protein [Streptomyces sp. CB03234]	WP_079277982.1	-	-	-
10	CstA	636	Methyltransferase	class I SAM-dependent methyltransferase [Streptomyces monomycini]	WP_030021788.1	-	-	-
11	CstB	2214	Synthetase	hypothetical protein [Streptomyces monomycini]	WP_030021787.1	*pur 6*	0	52.98%
12	CstC	603	N-acetyltransferase	GNAT family N-acetyltransferase [Streptomyces monomycini]	WP_030021786.1	*pac*	1.00E-95	65.50%
13	CstD	810	Monophosphatase	histidinol-phosphatase [Streptomyces monomycini]	WP_030021785.1	*pur3*	3.00E-123	68.90%
14	CstE	690	N-Methyltransferase	methyltransferase [Streptomyces monomycini]	WP_030021784.1	*pur5*	1.00E-124	78.17%
15	CstF	1290	Aminotransferase	DegT/DnrJ/EryC1/StrS family aminotransferase [Streptomyces monomycini]	WP_078624438.1	*pur4*	0	77.78%
16	CstG	1002	Oxidoreductase	Gfo/Idh/MocA family oxidoreductase [Streptomyces monomycini]	WP_275127596.1	*pur10*	6.00E-145	61.29%
17	CstH	516	NTP-pyrophosphohydrolase	NUDIX domain-containing protein [Streptomyces paromomycinus]	WP_246177904.1	*pur7*	1.00E-62	64.10%

Back to Contents

18	CstI	1434	Hydrolase	amidohydrolase [Streptomyces paromomycinus]	GCD48216.1	*napH*	5.00E-160	53.98%
19	Orf19	1236		threonine--tRNA ligase [Streptomyces sp. SID4946]	WP_210167982.1	-	-	-
20	Orf20	336		alkene reductase [Streptomyces monomycini]	WP_050502695.1	-	-	-

Back to Contents

Supplement S2. The expanded analysis of the *cst* BGC

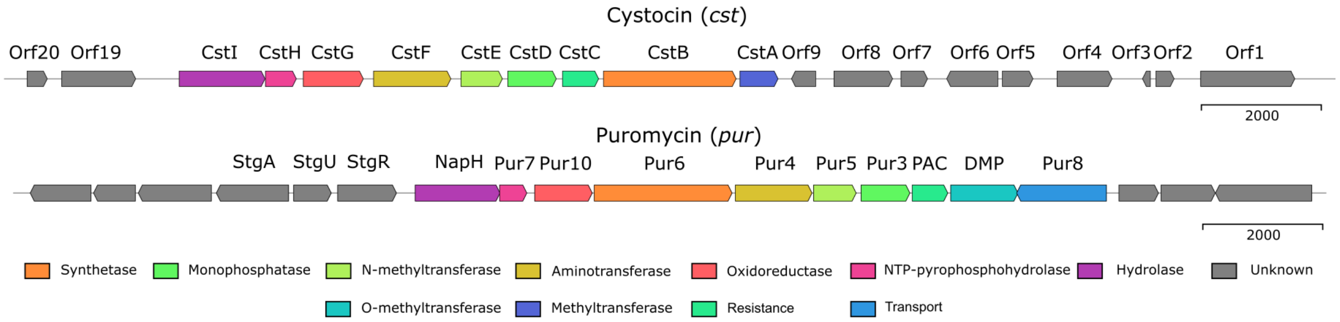

Although the cloning of the *pur* BGC indicated that *prg1* might be a regulatory gene within the *pur* cluster [7], further corroboration of its involvement is lacking. The *cst* BGC does not include any ORFs exhibiting similarity to either the *mtdA* or *prg1* genes. Intriguingly, in the DNA fragment adjacent to the puromycin BGC in *Streptomyces alboniger*, three ORFs –*stgA*, *stgU*, and *stgR* – are present. These ORFs contain a helix-turn-helix motif but have been shown not to be involved in Puro biosynthesis [8]. In the *cst* BGC there is a gene, *orf6*, also possessing the same motif. However, the encoded protein displays no similarity to *stgU*, making it unlikely to participate in the regulation of Cst biosynthesis.

Resistance to Puro has been linked to the involvement of the transmembrane protein Pur8 [9] and the N-acetyltransferase PAC [10]. In the context of the A201A-related nucleoside antibiotic, resistance is mediated by the genes *ard2* and *ard1*, which encode an ABC-transporter and a phosphotransferase, respectively [11,12]. The *cst* BGC lacks homologues of the transmembrane protein Pur8, which is presumed to facilitate the transport of N-acetylpuromycin out of the cells. We speculate that the gene encoding the transport-mediating enzyme might be situated elsewhere in the genome, external to the *cst* BGC.

Back to Contents

The biosynthesis of Puro seems to proceed without any obvious regulation, a situation that has been similarly observed in other *Streptomyces* gene clusters. A study of *pur* cluster expression showed that Puro production is subjected to translational dependence on the *bldA* gene product, specifically the $tRNA^{Leu}$ molecule, due to the presence of a TTA codon within the amino-terminal coding region of the *pur10* and *pur6* genes [13]. This kind of regulation was proposed in the blasticidin S biosynthetic gene cluster as well, where the *blsM* gene contains a TTA codon that governs translation, relying on the cellular levels of the *bldA* gene product. This particular $tRNA^{Leu}$ molecule is known to synchronize antibiotic biosynthesis events with the developmental cycle in numerous *Streptomyces* species [14]. Interestingly, for A201A, the MtdA protein was shown to play a role in regulation [15]. Notably, the *ataP10* gene in the biosynthesis of A201A lacks TTA codon, which is found in the *pur10* gene and appears to contribute to the regulation of the *pur* cluster [11]. In the *cst* BGC, the homologues of *pur10* and *pur6* genes, *cstG* and *cstB*, similarly lack the TTA codon. This suggests that the regulation of Cst expression differs from that of Puro.

Back to Contents

Table S2. Susceptibility of WT. CstC and PAC-transformed eukaryotic HEK293T WT

Cell line	IC_{50}. µg/ml	
	Puro	Cst
HEK293T WT	0.2 ± 0.02	1 ± 0.08
HEK293T PAC	6 ± 6	± 15
HEK293T CstC	5 ± 9	> 100

Resistance coefficient for PAC gene 310 ± 1 (Puro) and 33 ± 12 (Cst).
Resistance coefficient for CstC gene 260 ± 20 (Puro) and over 87 (Cst).

By 72 h after adding the drug to the cell culture at concentrations 0.5 µg/mL and higher, cell death is already observed.

Back to Contents

Figure S2. Reporter strain screening of Cst and Puro

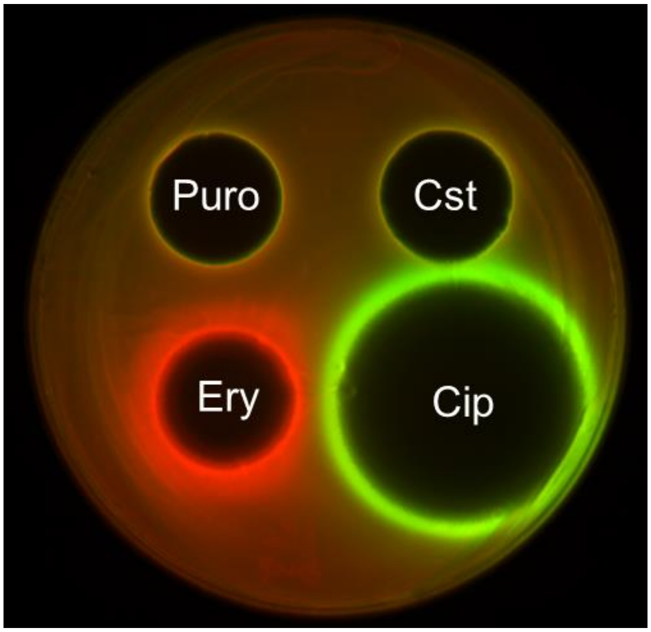

Evaluation of the antibacterial properties of the puromycin and cystocin on the hypersensitive strain of *E. coli* JW5503 (Δ*tolC*, with impaired efflux) pDualrep2, which consists of two fluorescent protein reporter genes, *turborfp* (induced by SOS-inducible *sulA* gene promoter) and *katushka2S* (regulated by the modified *trpL* attenuator sequence) [16]. There are shown Puro (20 µg) and Cst (20 µg) and control antibiotics («ery» is erythromycin (5 µg) and «cip» is ciprofloxacin (25 ng)

Two antibiotics were used as controls. The first was erythromycin, which disrupted protein biosynthesis in the bacterial cells and caused gene expression of the fluorescent protein Katushka2S (indicated by the red edge around the inhibition zone). The second control antibiotic was ciprofloxacin, which inhibited the work of gyrases and triggered expression of the fluorescent protein TurboRFP (indicated the green edge around the inhibition zone). For Cst and Puro inhibiting protein biosynthesis is marked by Katushka2S fluorescence (red edge).

Back to Contents

Supplement S3. BODIPY-labelled small peptides synthesis inhibition experiment

The experiment was performed as described previously, including hydrolysis with 1M $NaHCO_3$ prior to gel electrophoresis [17]. In case of aminonucleosides formation of modified truncated products can be anticipated (Figure S2-1).

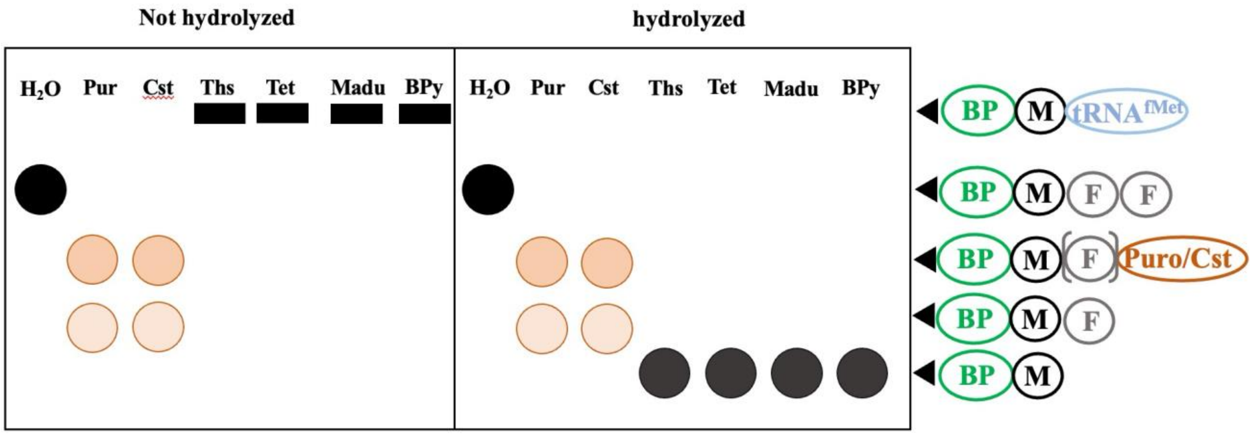

Figure S3-1. Schematic representation of the anticipated results in BODIPY-labelled small peptides inhibition experiment.

In line with the previous results for Puro [17], no truncated peptides were detected for Puro and Cst, presumably because the mobility of peptidyl-puromycin and peptidyl-cystocin, formed after transferring of the peptide to antibiotic molecule, have the opposite charge and thus different mobility than the BODIPY-labeled peptides, so that they did not enter into RNA PAGE. Alternatively, Puro/Cst coupling may lead to quenching of BODIPY fluorescence. The “anomalous” migration of puromycin in the PAGE gel deserves a separate discussion, since Puro causes the transfer of the growing peptide labeled with BODIPY onto itself, and it is impossible to predict its visualization on the gel in advance. Probably, Puro/Cst with the labeled peptide move in the direction opposite to the other peptides, therefore the “spots” observed on the gel for the products treated with Puro/Cst before and after the hydrolysis are less bright. Alternatively, the

Back to Contents

anticipated products containing Puro/Cst are not visible due to quenching of BODIPY fluorescence. Further the obtained results are provided (Figures S2-2, S2-3, S2-4).

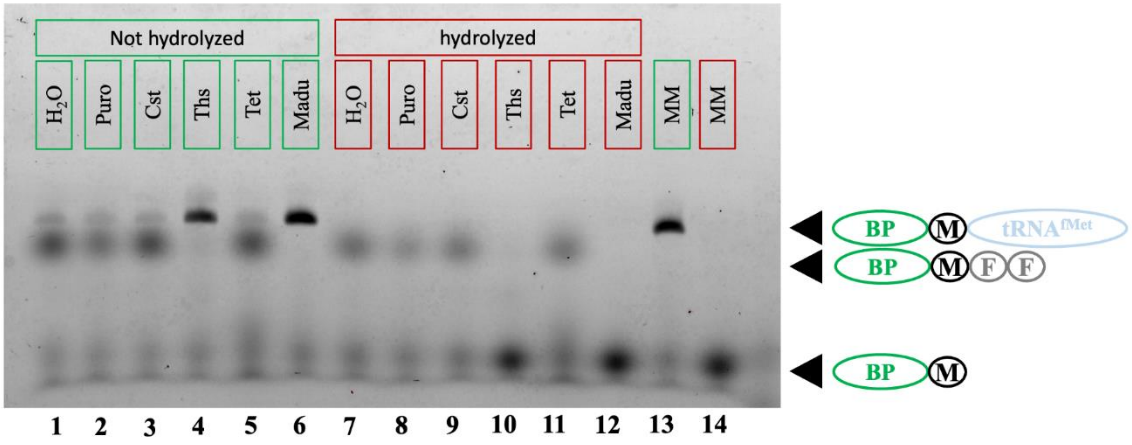

	Not hydrolyzed			
	water	Puro	Cst	Tet
Translation efficiency	1	0.8	1	0.69

Figure S3-2. Urea PAGE electrophoresis of MF2 synthesis under treatment with 0.05 mM of Cst and Puro. Ths – thiostrepton; Madu – madumycin; MM – Master Mix; Tet – tetracycline; BP – BODIPY.

Back to Contents

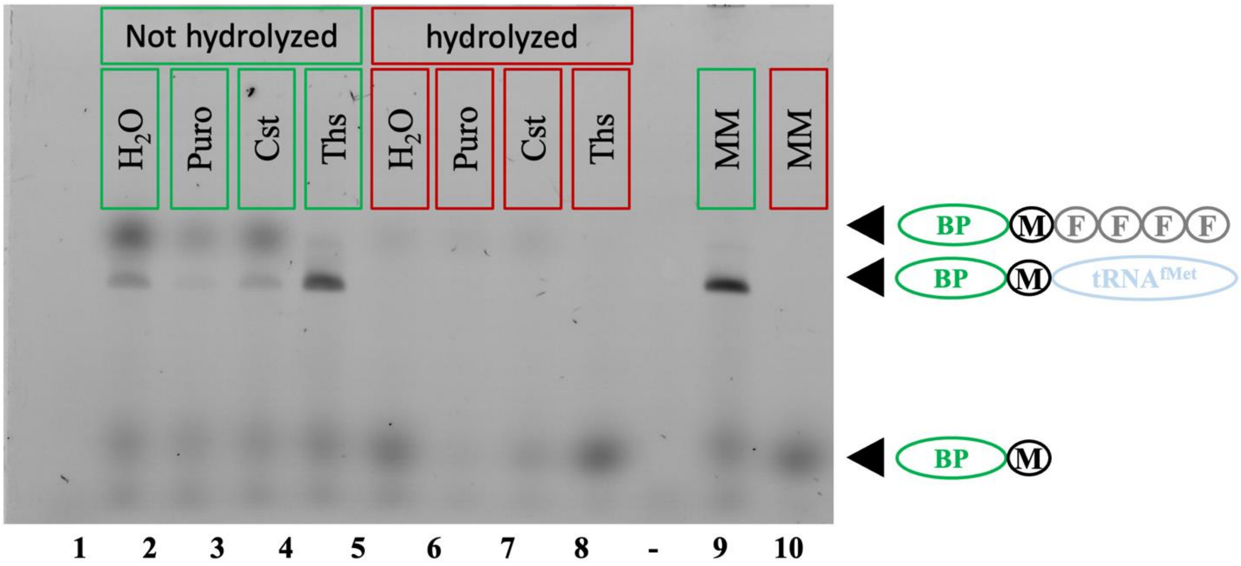

	Not hydrolyzed		
	water	Puro	Cst
Translation efficiency	1	0.39	0.67

Figure S3-3. PAGE electrophoresis of MF_4 synthesis under treatment with 0.05 mM of Cst and Puro. Ths – thiostrepton; Madu – madumycin; MM – Master Mix; Tet – tetracycline; BP – BODIPY.

Back to Contents

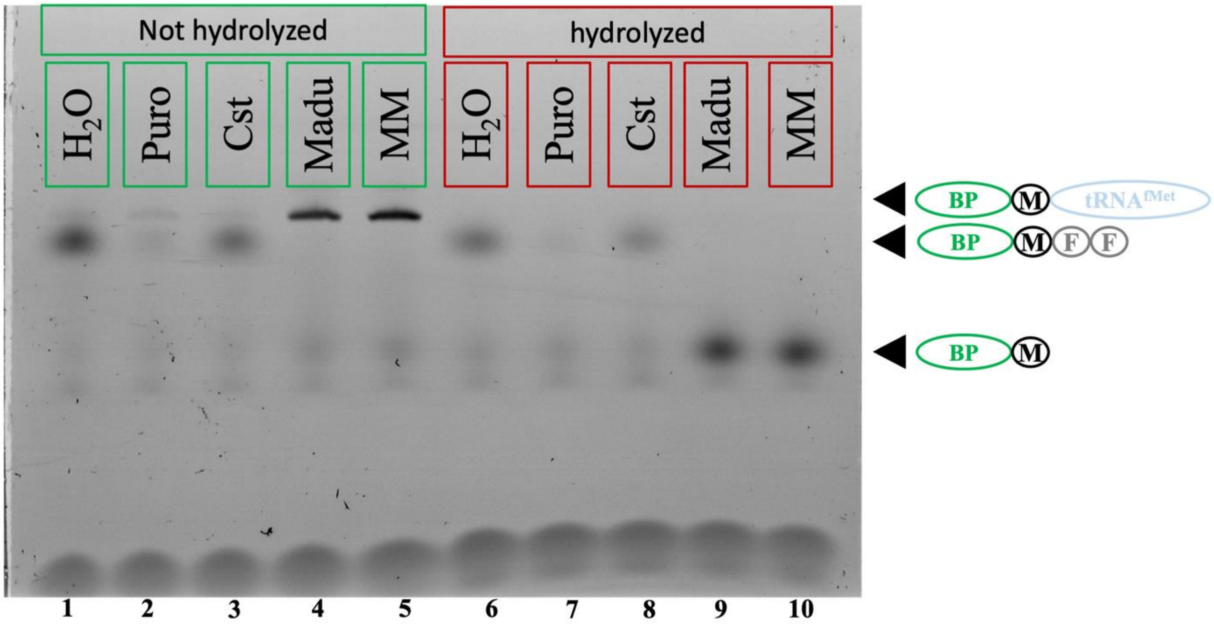

	Not hydrolyzed		
	water	Puro	Cst
Translation efficiency	1	0.38	0.71

Figure S3-4. PAGE electrophoresis of MF_4 synthesis under treatment with 4 mM of Cst and Puro. Ths – thiostrepton; Madu – madumycin; MM – Master Mix; Tet – tetracycline; BP – BODIPY.

Back to Contents

Supplement S4. MS analysis of small peptides synthesis inhibition reactions

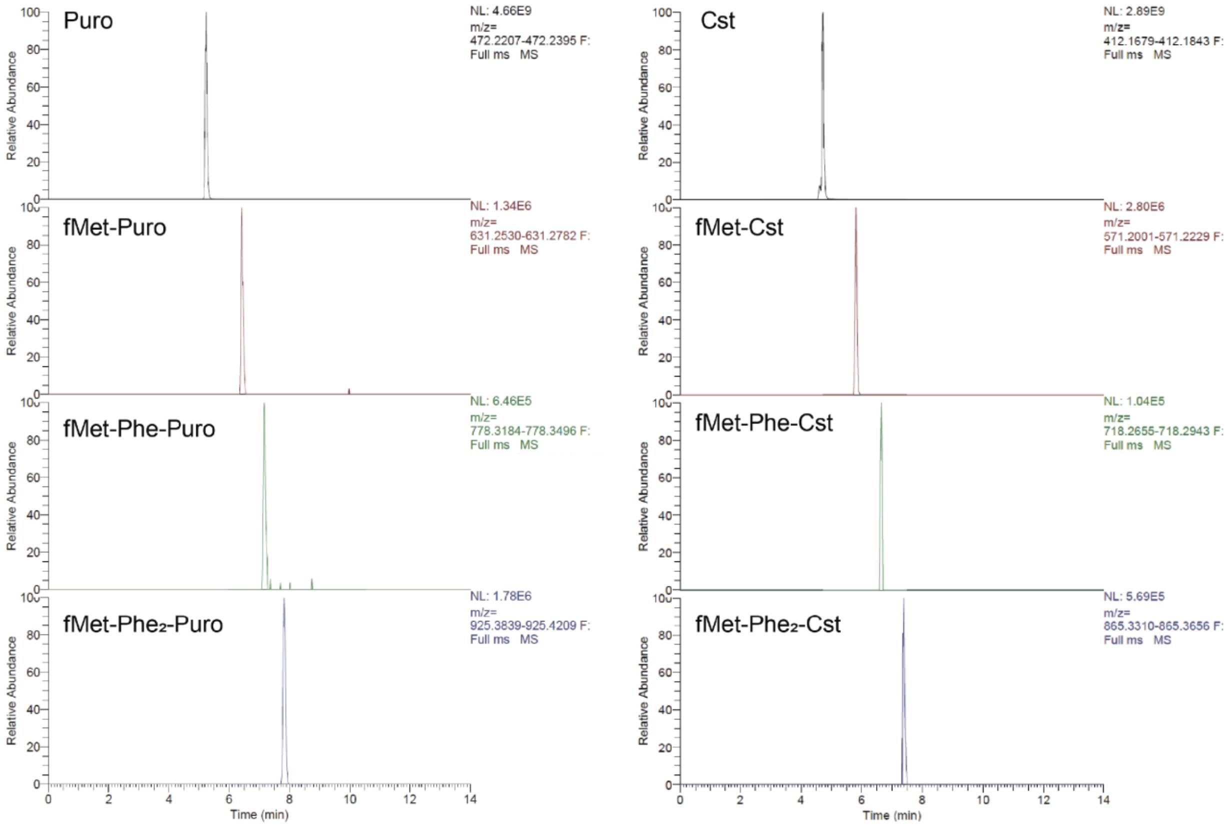

	Molecular formula	[M+H]+ calc	[M+H]+ found
Puro	C22H29N7O5	472.2301	472.2317
fMet-Puro	C28H38N8O7S	631.2656	631.2671
fMet-Phe-Puro	C37H47N9O8S	778.334	778.3362
fMet-Phe2-Puro	C46H56N10O9S	925.4024	925.4082
fMet-Phe3-Puro	C55H65N11O10S	1072.471	-
Cst	C16H25N7O4S	412.1770	412.1790
fMet-Cst	C22H34N8O6S2	571.2121	571.2125
fMet-Phe-Cst	C31H43N9O7S2	718.2805	718.2820
fMet-Phe2-Cst	C40H52N10O8S2	865.3489	865.3502
fMet-Phe3-Cst	C49H61N11O9S2	1012.417	-

Figure S4-1. A plot of extracted ion chromatograms (XICs) showing distinct peaks for cystocinilated and puromycinilated truncated peptides

Back to Contents

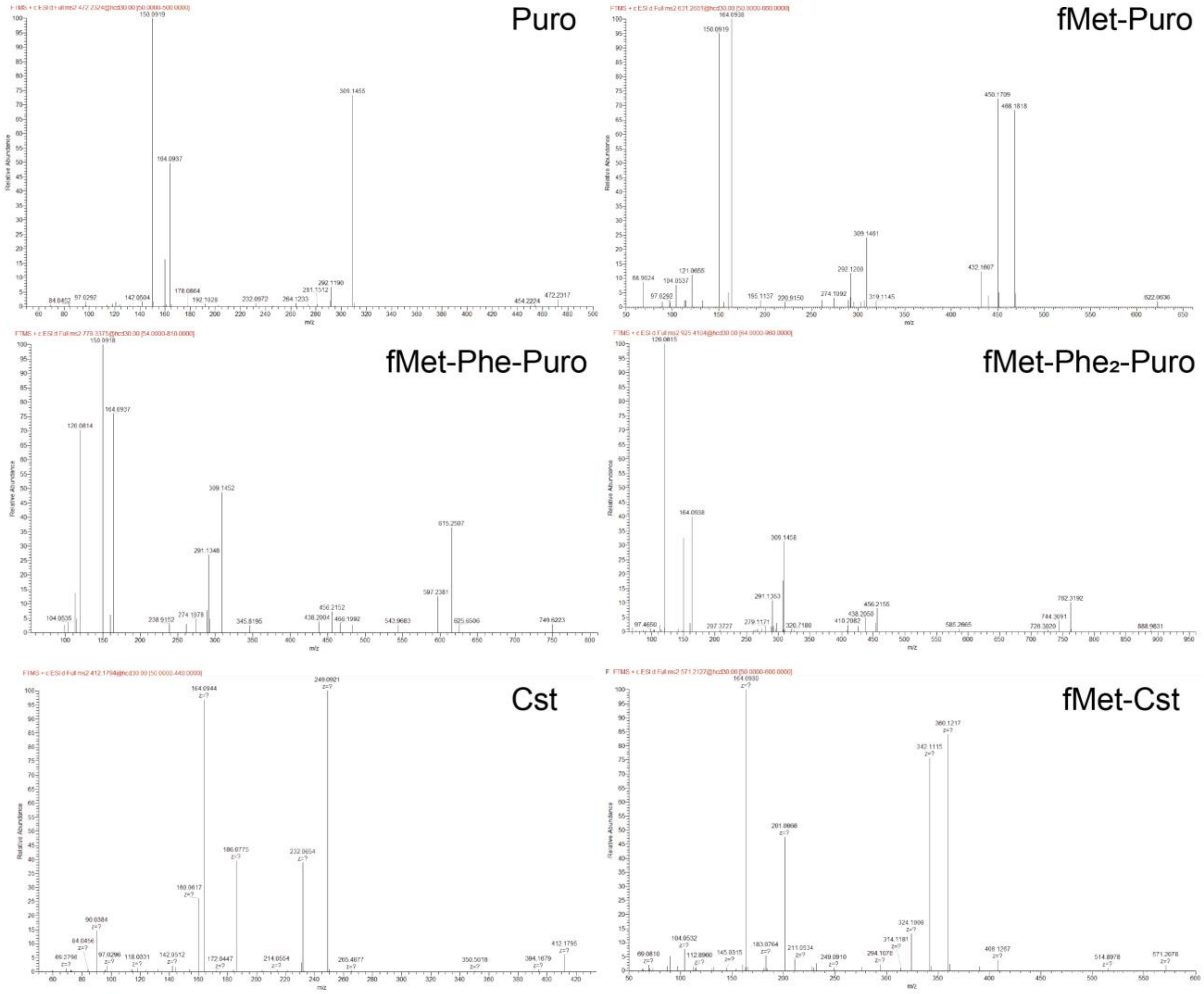

Figure S4-1. MS/MS spectra highlighting the fragmentation patterns of cystocinilated and puromycinilated truncated peptides

Back to Contents

Figure S3. HPLC-trace of the isolated cystocin fraction

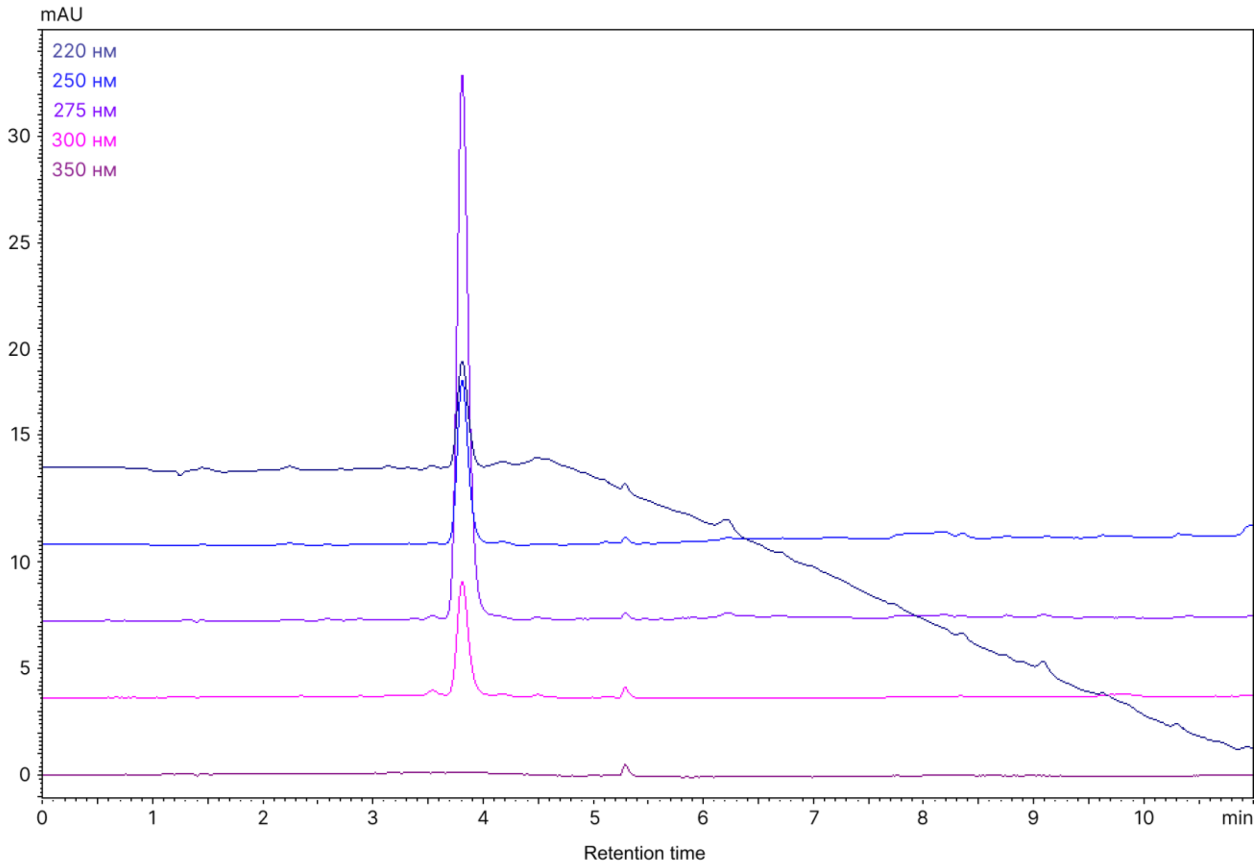

Pure component was analyzed by HPLC on RP column using Nexera X2 LC 30A instrument (Shimadzu) equipped with SPD-M20A detector. HPLC conditions were as following: column Agilent HC-C18(2) 4.6 x 150 mm. 5 µm. eluent: solvent A - 10 mM NH_4OAc. pH 5. solvent B – MeCN. gradient elution from 15 to 90 solvent B for 10 min. flow rate 1.5 ml/min. UV detection at 230. 250. 275. 300. 350 nm.

Back to Contents

Table S3. Sequences of templates and primers used for toeprinting assay

Template	Sequence
MF_2	ATGCATAATACGACTCACTATAGGGCTTAAGTATAAGGAGGAAAAC ATATGTTCTTCTAAGAGACGGACGAGAGCGGC
MF_4	ATGCATAATACGACTCACTATAGGGCTTAAGTATAAGGAGGAAAAC ATATGTTCTTCTTCTTCTAAGAGACGGACGAGAGCGGC
ErmCL	ACTAATACGACTCACTATAGGGAGTTTTATAAGGAGGAAAAAATAT GGGCATTTTTAGTATTTTTGTAATCAGCACAGTTCATTATCAACCAA ACAAAAAATAATAATAATAAAAAAAGTGATAGAATTCTATCGTTAA TAAGCAAAATTCATTATAACC
NV1	GGTTATAATGAATTTTGCTTATTAAC

Back to Contents

Supplemental references

1. Goris, J.; Konstantinidis, K.T.; Klappenbach, J.A.; Coenye, T.; Vandamme, P.; Tiedje, J.M. DNA–DNA Hybridization Values and Their Relationship to Whole-Genome Sequence Similarities. *International Journal of Systematic and Evolutionary Microbiology* **2007**, *57*, 81–91, doi:10.1099/ijs.0.64483-0.
2. Chun, J.; Oren, A.; Ventosa, A.; Christensen, H.; Arahal, D.R.; Da Costa, M.S.; Rooney, A.P.; Yi, H.; Xu, X.-W.; De Meyer, S.; et al. Proposed Minimal Standards for the Use of Genome Data for the Taxonomy of Prokaryotes. *International Journal of Systematic and Evolutionary Microbiology* **2018**, *68*, 461–466, doi:10.1099/ijsem.0.002516.
3. Konstantinidis, K.T.; Ramette, A.; Tiedje, J.M. Toward a More Robust Assessment of Intraspecies Diversity, Using Fewer Genetic Markers. *Appl Environ Microbiol* **2006**, *72*, 7286–7293, doi:10.1128/AEM.01398-06.
4. Hu, S.; Li, K.; Zhang, Y.; Wang, Y.; Fu, L.; Xiao, Y.; Tang, X.; Gao, J. New Insights Into the Threshold Values of Multi-Locus Sequence Analysis, Average Nucleotide Identity and Digital DNA–DNA Hybridization in Delineating Streptomyces Species. *Front. Microbiol.* **2022**, *13*, 910277, doi:10.3389/fmicb.2022.910277.
5. Krassilnikov, N.A.; Koveshnikov, A.D. *Actinomyces Tumemacerans* n. Sp. – a New Species Inducing Disintegration of Tumors in Plant. *Mikrobiologiia* **1962**, *31*, 589–594.
6. Pridham, T.G. New Names and New Combinations in the Order Actinomycetales Buchanan 1917. *Bull. United States Dep. Agric.* **1970**, *1424*, 1–55.
7. Lacalle, R.A.; Tercero, J.A.; Jiménez, A. Cloning of the Complete Biosynthetic Gene Cluster for an Aminonucleoside Antibiotic, Puromycin, and Its Regulated Expression in Heterologous Hosts. *The EMBO Journal* **1992**, *11*, 785–792, doi:10.1002/j.1460-2075.1992.tb05112.x.
8. Tercero, J.A.; Espinosa, J.C.; Jiménez, A. StgR, a New *Streptomyces Alboniger* Member of the LysR Family of Transcriptional Regulators. *Mol Gen Genet* **1998**, *259*, 475–483, doi:10.1007/s004380050838.
9. Tercero, J.A.; Lacalle, R.A.; Jimenez, A. The *Pur8* Gene from the *Pur* Cluster of *Streptomyces Alboniger* Encodes a Highly Hydrophobic Polypeptide Which Confers Resistance to Puromycin. *Eur J Biochem* **1993**, *218*, 963–971, doi:10.1111/j.1432-1033.1993.tb18454.x.
10. Lacalle, R.A.; Pulido, D.; Vara, J.; Zaiacaín, M.; Jiménez, A. Molecular Analysis of the *Pac* Gene Encoding a Puromycin N-Acetyl Transferase from *Streptomyces Alboniger*. *Gene* **1989**, *79*, 375–380, doi:10.1016/0378-1119(89)90220-5.
11. Saugar, I.; Sanz, E.; Rubio, M.Á.; Espinosa, J.C.; Jiménez, A. Identification of a Set of Genes Involved in the Biosynthesis of the Aminonucleoside Moiety of Antibiotic A201A from *Streptomyces Capreolus*: Aminonucleoside A201A Biosynthetic Genes. *European Journal of Biochemistry* **2002**, *269*, 5527–5535, doi:10.1046/j.1432-1033.2002.03258.x.
12. Barrasa, M.I.; Tercero, J.A.; Jimenez, A. The Aminonucleoside Antibiotic A201A Is Inactivated by a Phosphotransferase Activity from *Streptomyces Capreolus* NRRL 3817, the Producing Organism. Isolation and Molecular Characterization of the Relevant Encoding Gene and Its DNA Flanking Regions. *Eur J Biochem* **1997**, *245*, 54–63, doi:10.1111/j.1432-1033.1997.00054.x.

Back to Contents

13. Tercero, J.A.; Espinosa, J.C.; Jiménez, A. Expression of the *Streptomyces Alboniger Pur* Cluster in *Streptomyces Lividans* Is Dependent on the *bldA* -Encoded tRNA ^{Leu}. *FEBS Letters* **1998**, *421*, 221–223, doi:10.1016/S0014-5793(97)01564-0.
14. Cone, M.C.; Yin, X.; Grochowski, L.L.; Parker, M.R.; Zabriskie, T.M. The Blasticidin s Biosynthesis Gene Cluster from *Streptomyces Griseochromogenes*: Sequence Analysis, Organization, and Initial Characterization. *ChemBioChem* **2003**, *4*, 821–828, doi:10.1002/cbic.200300583.
15. Zhu, Q.; Li, J.; Ma, J.; Luo, M.; Wang, B.; Huang, H.; Tian, X.; Li, W.; Zhang, S.; Zhang, C.; et al. Discovery and Engineered Overproduction of Antimicrobial Nucleoside Antibiotic A201A from the Deep-Sea Marine Actinomycete *Marinactinospora Thermotolerans* Scsio 00652. *Antimicrob Agents Chemother* **2012**, *56*, 110–114, doi:10.1128/AAC.05278-11.
16. Osterman, I.A.; Komarova, E.S.; Shiryaev, D.I.; Korniltsev, I.A.; Khven, I.M.; Lukyanov, D.A.; Tashlitsky, V.N.; Serebryakova, M.V.; Efremenkova, O.V.; Ivanenkov, Y.A.; et al. Sorting Out Antibiotics' Mechanisms of Action: A Double Fluorescent Protein Reporter for High-Throughput Screening of Ribosome and DNA Biosynthesis Inhibitors. *Antimicrob Agents Chemother* **2016**, *60*, 7481–7489, doi:10.1128/AAC.02117-16.
17. Marina, V.I.; Bidzhieva, M.; Tereshchenkov, A.G.; Orekhov, D.; Sagitova, V.E.; Sumbatyan, N.V.; Tashlitsky, V.N.; Ferberg, A.S.; Maviza, T.P.; Kasatsky, P.; et al. An Easy Tool to Monitor the Elemental Steps of in Vitro Translation via Gel Electrophoresis of Fluorescently Labeled Small Peptides. *RNA* **2024**, *30*, 298–307, doi:10.1261/rna.079766.123.

Back to Contents
